# Methadone for Palliative Care Providers: A Case-Based Flipped Classroom Module for Faculty and Fellows

**DOI:** 10.15766/mep_2374-8265.11172

**Published:** 2021-07-26

**Authors:** Mollie Alexandra Biewald, Bethann Scarborough, Elizabeth Lindenberger

**Affiliations:** 1 Assistant Professor, Brookdale Department of Geriatrics and Palliative Medicine, Icahn School of Medicine at Mount Sinai; 2 Associate Professor, Brookdale Department of Geriatrics and Palliative Medicine, Icahn School of Medicine at Mount Sinai

**Keywords:** Methadone, Pain Management, Hospice and Palliative Medicine, Pain Medicine, Clinical/Procedural Skills Training, Flipped Classroom, Opioids

## Abstract

**Introduction:**

Methadone is an effective medication for treating pain and has unique characteristics that require specialized knowledge to prescribe safely. Palliative care providers use methadone for analgesia in patients with a wide range of prognoses, goals of care, and comorbid conditions. New consensus guidelines for methadone use released in 2019 by the American Academy of Hospice and Palliative Medicine provide guidance for safe use in patients who have potentially life-limiting illnesses. A needs assessment of palliative care fellows and faculty at our institution highlighted lack of knowledge and confidence with regard to prescribing methadone.

**Methods:**

We created a virtual, flipped classroom, interactive learning module intended for palliative care fellows and practicing clinicians that emphasized updated practice recommendations. Participants took a pretest, reviewed an article and lecture, and completed practice cases prior to an interactive session conducted via videoconference. Following the session, participants completed a posttest to assess knowledge and confidence regarding the learning objectives.

**Results:**

A total of 28 clinicians at the fellow and faculty/staff levels completed the intervention during two sessions in 2020. Self-reported confidence in all educational objectives improved following the intervention. Participants demonstrated improved skill in calculating methadone doses, converting between modes of drug administration, and identifying safety guidelines during and after the intervention.

**Discussion:**

Following the intervention, participants reported improved confidence and demonstrated improved skills in prescribing methadone for pain. Additional benefits of this training model include ease of implementation, engaging format, and time and resource efficiency given its virtual format.

## Educational Objectives

By the end of this activity, learners will be able to:

1. Select appropriate candidates for methadone therapy based on comorbidities and psychosocial issues.
2. Calculate starting doses of methadone based on a patient's prior opioid use.
3. Choose degree of cardiac monitoring based on a patient's prognosis and goals of care.

## Introduction

Methadone is a powerful tool for managing pain in patients with serious illness. Given the drug's activity at multiple receptors and unpredictable pharmacokinetics, it has many unique pharmacologic properties and requires expertise to prescribe safely. The Centers for Disease Control and Prevention report that while methadone accounted for only 1% of opioid prescriptions in 2014, it was responsible for 23% of prescription opioid deaths.^1^ In 2015, methadone was the cause of more than 5,500 hospitalizations in the United States.^2^ Despite risks, for many patients with pain due to serious illness, methadone can dramatically improve symptom control and quality of life.^3^ New guidelines authored by McPherson and colleagues and published in 2019 provide a framework for palliative care providers to prescribe methadone safely for pain.^4^

Fellows in our palliative care program identified methadone as a topic they were motivated to learn about. In 2019, we conducted a needs assessment of 14 physician and nurse practitioner fellows at our institution and 10 interdisciplinary faculty members who supervised trainees. We found that 93% of fellows and 100% of faculty felt methadone should be covered in an ambulatory palliative care curriculum. Notably, fellows identified methadone as the topic they felt most unprepared to handle in clinical practice. Faculty members agreed with this assessment, rating methadone among the two highest topics they thought fellows were not prepared to manage. The reason for this perceived lack of comfort with methadone may have been in part due to the wide practice variation prior to release of the 2019 guidelines, including at least five accepted conversion charts to calculate doses.^5^ Previously released guidelines for methadone use did not address clinical issues relevant to many patients treated by palliative care providers, including variations in prognosis, goals of care, and comorbid organ dysfunction.^6^

The release of new guidelines specific to palliative care provided an opportunity to update learners as well as experienced clinicians. This learning module is intended for physicians and advanced practice providers, at the residency level and beyond, who intend to prescribe methadone for analgesia in the inpatient or outpatient setting. The module covers patient selection, calculation of safe and effective dosing, and choosing a level of surveillance for adverse effects based on a patient's goals of care.

We are aware of no existing resources that integrate the new guidelines for methadone in palliative care into clinical practice. This module is appropriate for palliative care clinicians with a wide range of prior experience, whether still in training or already in practice for many years. Because of the complexity of prescribing methadone for pain with regard to the pharmacologic properties and safety concerns discussed above, we hope this resource will be a useful tool for educators and learners at multiple institutions. This publication builds on multiple existing *MedEdPORTAL* resources that teach safe opioid prescribing through interactive cases, such as the road map for opioid use by Lester, Remolana, Sandhu, and Scott^7^ and recent work by Sagin and colleauges^8^ teaching an interdisciplinary approach to pain management to students. Our workshop was created in response to learners’ self-identified need for training on this topic, similar to the session by Vettese, Thati, and Roxas on outpatient opioid prescribing that was developed in response to residents’ reported learning needs.^9^ Finally, our module is intended to be used as a faculty development workshop prior to implementation with postgraduate learners, like the interactive workshop by Gaufberg, Barnes, Albanese, and Cohen for residency clinic preceptors on responding to opioid requests for chronic pain.^10^ Our learning module offers palliative care clinicians with diverse levels of experience an opportunity to build skill and confidence using a challenging and unique medication safely.

To meet the methadone training needs of our palliative care clinicians, we developed a flipped classroom training module that included a prework video and reading material followed by virtual live teaching that was entirely interactive. Because the methadone guidelines covered in the module were new to all clinicians regardless of experience level, we chose to include fellows and practicing clinicians simultaneously.

The flipped classroom model, in which students engage in asynchronous independent learning prior to class then use synchronous class time for interaction and critical thinking, is associated with improved learner engagement and, in some studies, increased knowledge acquisition.^11,12^ Given the COVID-19 pandemic, virtual training has become increasingly important and also adds potential advantages in terms of learner ease of attendance, resource efficiency, and scalability.

## Methods

We held this virtual workshop twice in the fall of 2020. The workshop was open to all clinicians in the Department of Geriatrics and Palliative Medicine, including physician fellows, practicing physicians, and nurse practitioners. Participants attended the entire session virtually due to the COVID-19 pandemic. To allow maximum participation, we held two virtual sessions: one during fellows’ weekly academic block and one during a recurring departmental meeting time. All participants were assumed to have existing basic understanding of opioid pharmacology, opioid conversions, and pain assessment based on their required participation in prior sessions covering these topics. We used a flipped classroom model to maximize learning and engagement among a group of clinicians with diverse training backgrounds within palliative care and levels of experience with prescribing methadone.^12^

One week prior to the session, participants were sent an email with links to the pretest (Appendix A), the new published guidelines,^4^ and a 25-minute slide-based lecture reviewing highlights of the new guidelines and offering a detailed, step-by-step explanation of how to perform a conversion problem (Appendix B). The lecture was originally recorded as an MP4 but is presented here in the form of presentation slides with notes to maximize generalizability at other sites. A one-page cheat-sheet summary reviewing conversions and titration (Appendix C) and a set of practice cases (Appendix D) were included. Participants were instructed to complete the pretest, review the guidelines and lecture, and then complete the cases prior to the interactive session.

During the interactive session held via the Zoom videoconference platform, we divided the participants into breakout rooms with a maximum of six participants each. We asked participants to discuss as a small group their answers to cases they had completed prior to the session. They were given 8 minutes to discuss each case. Upon returning to the large group, participants shared their answers to the cases via polling, and poll data were shared with the group. A representative from each small group described highlights from the group's discussion and how the case had been approached. We reviewed the answers to each question based on the teaching guide (Appendix E), which was given to participants at the end of the session. Throughout the session, participants were encouraged to ask questions and share practical tips from their own previous experience prescribing methadone. The day after the session, we sent participants an email with a link to the session posttest (Appendix F). One week later, we sent a reminder email asking that they complete the posttest. An answer guide to the posttest was included (Appendix G).

We obtained institutional review board exemption for analysis of our workshop. Based on principles of survey design in medical education, we designed a survey to assess participants’ level of confidence in performing the educational objectives and their ability to safely prescribe methadone.^13^ Because our goal in creating this session was to improve skill in use of a complex medication, the pre- and posttests required participants to perform mathematical opioid conversions and answer knowledge questions about safety guidelines. Using videoconference software for the interactive component of the session allowed us to collect additional data from participants’ answers to poll questions.

## Results

We implemented the workshop with physician and nurse practitioner fellows and practicing clinicians during two separate 1-hour interactive videoconference workshops in October and December 2020. The session was open to all physicians and nurse practitioners in the Department of Geriatrics and Palliative Medicine. A total of 34 participants completed the pretest, of whom 16 were in fellowship and 18 were practicing clinicians. Physicians comprised the majority of both groups: 81% of fellows and 67% of practicing clinicians. A minority of fellows (13%) reported prior training about methadone, while the majority of practicing clinicians (78%) had some prior training. Participants were asked to estimate the number of patients they had cared for who had been prescribed methadone for pain. Fellows reported caring for an average of 2.9 patients using methadone versus practicing clinicians, who reported an average of 3.6 such patients over the past year. A total of 28 participants attended the interactive workshop, and 13 completed the posttest between 1 and 7 days following the session (46% response rate).

Participants in both groups rated the importance of being able to initiate and titrate methadone as highly important to their practice (Table 1). Self-reported confidence in selecting appropriate candidates for methadone, calculating starting doses, and performing cardiac monitoring were lower among fellows than practicing clinicians, and the average confidence rating increased in both groups on all skills following the intervention.

**Table 1.**
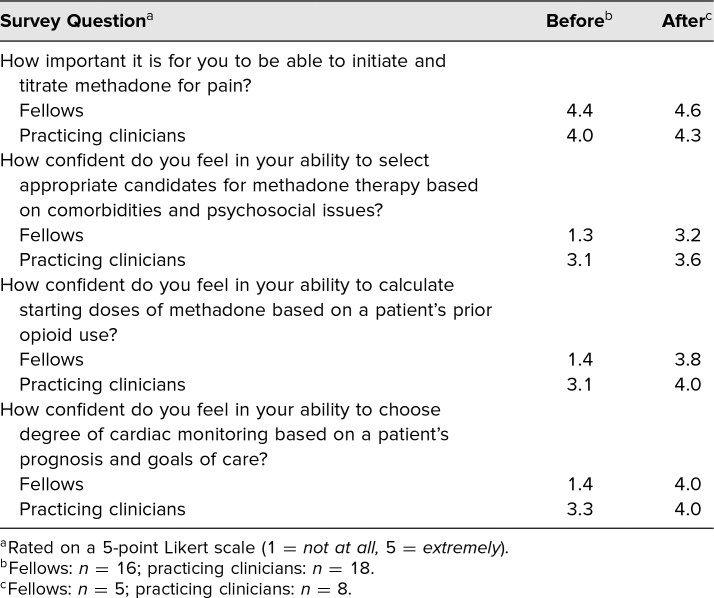
Average Survey Scores Immediately Before and Within 1 Week After Intervention

In order to assess participants' skill in performing the educational objectives for the session, five knowledge questions were included in the pre- and posttests (Table 2). Practicing clinicians answered a higher percentage of questions correctly on the pretest compared to fellows, and both groups improved in all five topics after the intervention.

**Table 2.**
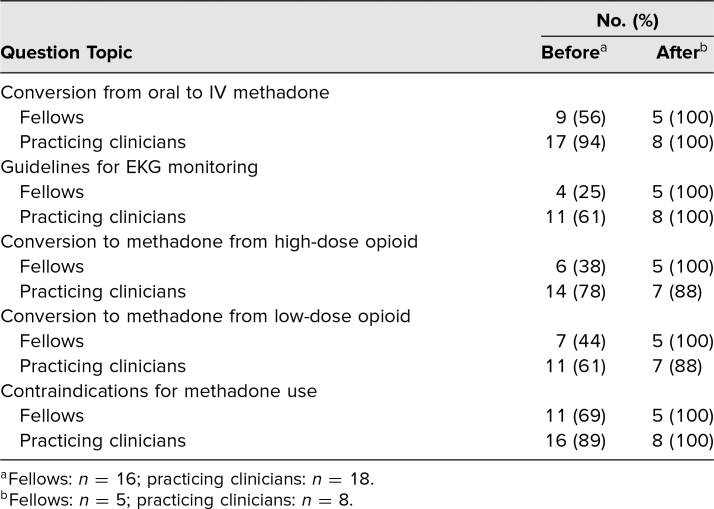
Correct Answers on Knowledge Questions Before and Within 1 Week After Intervention

## Discussion

Our workshop on methadone for palliative care providers is an interactive and easily implemented session that addresses practical concerns about the use of a complex and effective treatment for pain. The content is taught in a flipped classroom model in which participants review new guidelines and attempted practice cases prior to the session, allowing the hour-long synchronous component of the workshop to focus on discussion. We used the release of new guidelines specific to palliative care to address a knowledge gap identified by fellows, as well as to update our faculty to ensure that up-to-date information would be taught to learners in clinical practice. Following the session, participants demonstrated improved skill and confidence in the educational objectives discussed.

An advantage of our session is the short, 1-hour time frame emphasizing interactive, synchronous learning. The short duration and virtual formal likely allowed more faculty to attend despite conflicting responsibilities. The asynchronous, precourse component could be completed at learners’ time convenience, and with this preparation, all synchronous time could be spent in interactive case-based problem-solving work. This approach was well received by participants, with free-text comments on the posttest including “I really appreciate the practice problems because it helps me feel more comfortable for when I actually have to prescribe it.” Although the short 1-hour time presented advantages, it also showed limitations. Evaluations from multiple participants requested a second hour for additional discussion and practice; based on this feedback, an additional follow-up workshop has been added for further practice.

A study limitation was the low posttest response rate, which left open the possibility of nonresponse bias: for instance, that those who responded were those who were already more skilled or knowledgeable. Another limitation was that the posttest was completed soon after the training completion and therefore did not capture skill-level changes retained weeks or months after the session. We hope to study long-term retention of these skills in future work. While the virtual platform provided some advantages, it was difficult to assess whether all participants were engaged and following the material. In a prior, in-person version of the interactive session, participants were divided into small groups in a conference room, and the facilitators could circulate through the room to answer questions and assess engagement. In addition, the asynchronous component of the module required a substantial amount of independent preparation, which may have limited the number of participants who completed the session. Note that 34 participants completed the pretest, while only 28 participated in an interactive session. We provided the physician fellows with 1 hour of protected time prior to the interactive session to complete the precourse component and in the future would recommend doing the same, if possible, for all participants.

Participants in the session commented that the flipped classroom format, structured discussion, and small-group cases were an effective and engaging way to learn and practice a new clinical skill. Evaluations from practicing clinicians as well as fellows requested that similarly structured sessions be held to cover additional topics in palliative care including opioid use disorder, use of transdermal fentanyl patches, and managing nonpain symptoms. Holding mixed sessions for practicing clinicians and fellows was well received by both groups of learners as a way to implement new practice guidelines, and while the two groups demonstrated different levels of skill and confidence on the pretest, both groups showed improvement after the session. We plan to use this framework as a way to review ongoing updates in clinical palliative care. If further related guidelines are released, this module could be easily updated to reflect changes. Lastly, while our 1-hour synchronous methadone training can be done in person, our study demonstrates the effectiveness of a virtual teaching model, which has added advantages of convenience, resource efficiency, and scalability.

## Appendices

Methadone Pretest.docxMethadone for Palliative Providers Slides.pptxMethadone Conversions and Titration Card.pdfMethadone Cases.docxMethadone Cases Teaching Guide.docxMethadone Posttest.docxMethadone Posttest Answer Key.docx *All appendices are peer reviewed as integral parts of the Original Publication.*

## References

[1] Faul M, Bohm M, Alexander C. Methadone prescribing and overdose and the association with Medicaid preferred drug list policies—United States, 2007–2014. MMWR Morb Mortal Wkly Rep. 2017;66(12):320–323. 10.15585/mmwr.mm6612a2 28358791 PMC5657959

[2] Centers for Disease Control and Prevention. Annual Surveillance Report of Drug-Related Risks and Outcomes—United States, 2017. Centers for Disease Control and Prevention, U.S. Department of Health and Human Services; 2017. Accessed May 24, 2021. https://www.cdc.gov/drugoverdose/pdf/pubs/2017-cdc-drug-surveillance-report.pdf

[3] McPherson ML. Demystifying Opioid Conversion Calculations: A Guide for Effective Dosing. 2nd ed. American Society of Health-System Pharmacists; 2018.

[4] McPherson ML, Walker KA, Davis MP, et al. Safe and appropriate use of methadone in hospice and palliative care: expert consensus white paper. J Pain Symptom Manage. 2019;57(3):635–645.E4. 10.1016/j.jpainsymman.2018.12.001 30578934

[5] McPherson ML. Demystifying Opioid Conversion Calculations: A Guide for Effective Dosing. American Society of Health-System Pharmacists; 2009.

[6] Chou R, Cruciani RA, Fiellin DA, et al. Methadone safety: a clinical practice guideline from the American Pain Society and College on Problems of Drug Dependence, in collaboration with the Heart Rhythm Society. J Pain. 2014;15(4):321–337. 10.1016/j.jpain.2014.01.494 24685458

[7] Lester P, Remolana R, Sandhu S, Scott J. Road map for opioid management in the inpatient setting: a structured approach to opioid selection and titration. MedEdPORTAL. 2016;12:10470. 10.15766/mep_2374-8265.10470 31008248 PMC6464480

[8] Sagin A, Kimberly SM, Farabelli JP, Schafer K, Kumar P, Uritsky TJ. Teaching pain management in serious illness in the era of the opioid epidemic: a team-based intervention. MedEdPORTAL. 2020;16:11006. 10.15766/mep_2374-8265.11006 33150202 PMC7597940

[9] Vettese TE, Thati N, Roxas R. Effective chronic pain management and responsible opioid prescribing: aligning a resident workshop to a protocol for improved outcomes. MedEdPORTAL. 2018;14:10756. 10.15766/mep_2374-8265.10756 30800956 PMC6342398

[10] Gaufberg E, Barnes HR, Albanese M, Cohen P. A faculty development workshop for primary care preceptors: helping your residents care for patients requesting opioids for chronic pain. MedEdPORTAL. 2011;7:8396. 10.15766/mep_2374-8265.8396

[11] Gillette C, Rudolph M, Kimble C, Rockich-Winston N, Smith L, Broedel-Zaugg K. A meta-analysis of outcomes comparing flipped classroom and lecture. Am J Pharm Educ. 2018;82(5):6898. 10.5688/ajpe6898 30013248 PMC6041496

[12] Chen F, Lui AM, Martinelli SM. A systematic review of the effectiveness of flipped classrooms in medical education. Med Educ. 2017;51(6):585–597. 10.1111/medu.13272 28488303

[13] Sullivan GM, Artino AR Jr. How to create a bad survey instrument. J Grad Med Educ. 2017;9(4):411–415. 10.4300/JGME-D-17-00375.1 28824750 PMC5559231

