# Supplementary material for: Methadone for Palliative Care Providers: A Case-Based Flipped Classroom Module for Faculty and Fellows
Source: MedEdPORTAL. 2021 Jul 26;17:11172. doi: 10.15766/mep_2374-8265.11172 (PMC8310899; doi:10.15766/mep_2374-8265.11172)
Supplement: Supplementary file 1 — Methadone Pretest.docxMethadone for Palliative Providers Slides.pptxMethadone Conversions and Titration Card.pdfMethadone Cases.docxMethadone Cases Teaching Guide.docxMethadone Posttest.docxMethadone Posttest Answer Key.docx [file mep_2374-8265.11172-s001.zip › A. Methadone Pretest.docx]

Methadone for Palliative Care Providers: Pre-Workshop Survey

1. What is your role?

[] MD

[] NP
[] Other: _________________________

2. Are you currently in fellowship?

[] Yes

[] No (number of years post-training): ____

3. Have you had prior training on use of methadone in palliative care?

[] No

[] Yes (please describe): _________________________________________

4. In the past year, I have cared for __ patients who were prescribed methadone for pain
[] 0
[] 1-2
[] 3-5
[] 6-10
[] Other: _______

5. How important it is for you to be able to initiate and titrate methadone for pain?

Not at all slightly moderately quite extremely

6. How confident do you feel in your ability to:

1. Select appropriate candidates for methadone therapy based on comorbidities and psychosocial issues

Not at all slightly moderately quite extremely

1. Calculate starting doses of methadone based on a patient’s prior opioid use

Not at all slightly moderately quite extremely

1. Choose degree of cardiac monitoring based on a patient’s prognosis and goals of care

Not at all slightly moderately quite extremely

Please answer the following questions about methadone:

7. Your patient currently takes morphine extended release 300mg twice a day. Convert this to methadone. What is the highest starting dose of methadone you would recommend?

1. 1mg PO q12h
2. 2.5mg PO q8h
3. 5mg PO q8h
4. 10mg PO q8h

8. Your patient currently takes long acting oxycodone 10mg q12h and oxycodone 5mg PO q4h prn. She took 2 prns in the past day. Convert this to methadone. What is the highest starting dose you would recommend?

1. 1mg PO q12h
2. 2.5mg PO q8h
3. 5mg PO q8h
4. 10mg PO q8h

9. Your patient takes methadone 5mg PO q8h around the clock. He can no longer tolerate PO. What is the equivalent IV methadone regimen?

1. 1mg IV q8h
2. 2.5mg IV q8h
3. 5mg IV q8h
4. 10mg IV q8h

10. For a patient with life-prolonging goals, when after initiating methadone for pain should you review an EKG to assess QTc?

1. After 24hr
2. After 3-5 days
3. After 1-2 weeks
4. After 2-4 weeks

11. Which comorbidity is not a contraindication to prescribing methadone for pain?

a. End stage renal disease on hemodialysis

b. Congenital prolonged QTc

c. Active opioid use disorder

d. Sleep apnea and active benzodiazepine use
