# Supplementary material for: Methadone for Palliative Care Providers: A Case-Based Flipped Classroom Module for Faculty and Fellows
Source: MedEdPORTAL. 2021 Jul 26;17:11172. doi: 10.15766/mep_2374-8265.11172 (PMC8310899; doi:10.15766/mep_2374-8265.11172)
Supplement: Supplementary file 1 — Methadone Pretest.docxMethadone for Palliative Providers Slides.pptxMethadone Conversions and Titration Card.pdfMethadone Cases.docxMethadone Cases Teaching Guide.docxMethadone Posttest.docxMethadone Posttest Answer Key.docx [file mep_2374-8265.11172-s001.zip › B. Methadone for Palliative Providers Slides.pptx]

## Slide 1
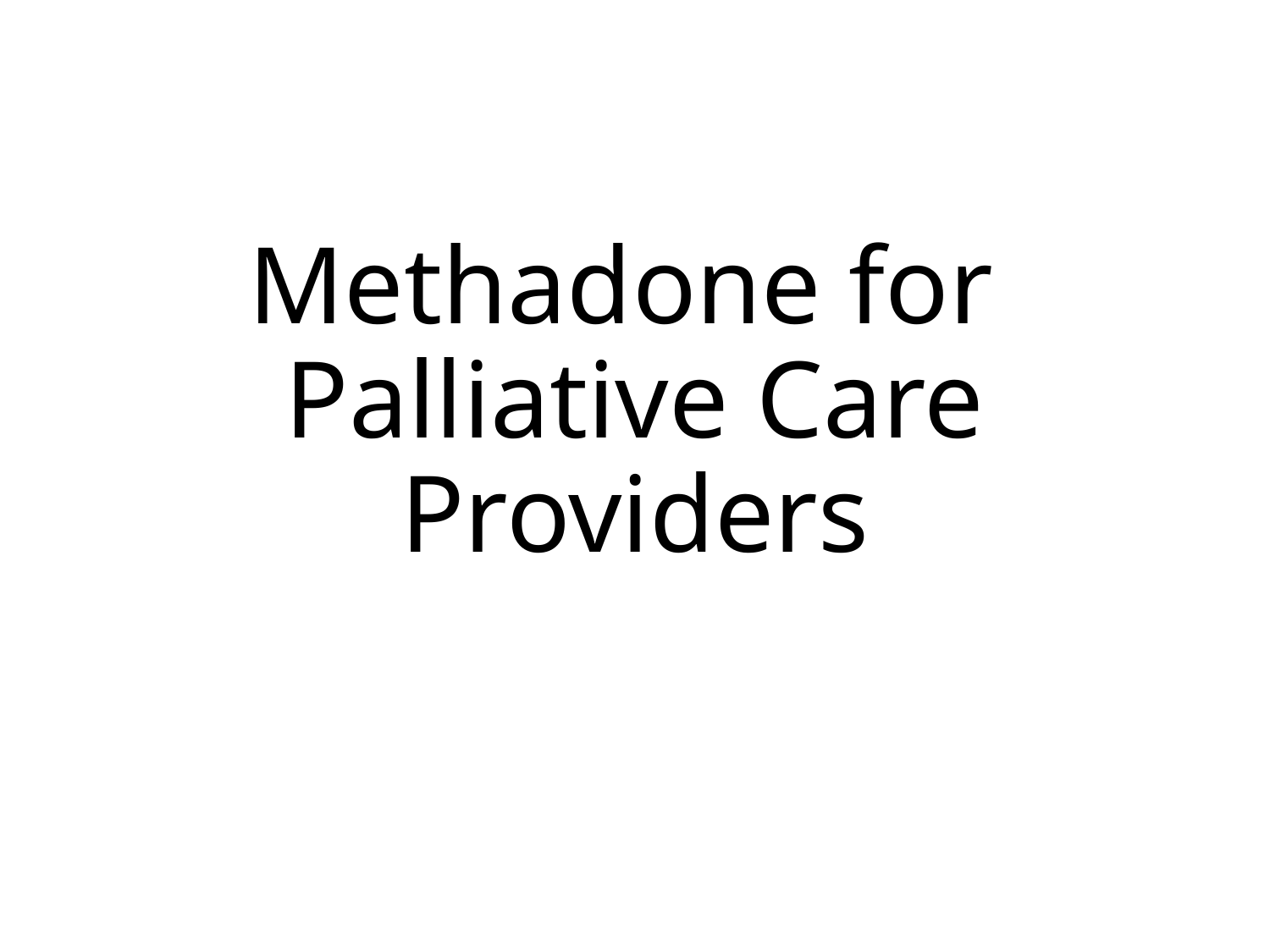

# Methadone for
Palliative Care Providers

## Slide 2
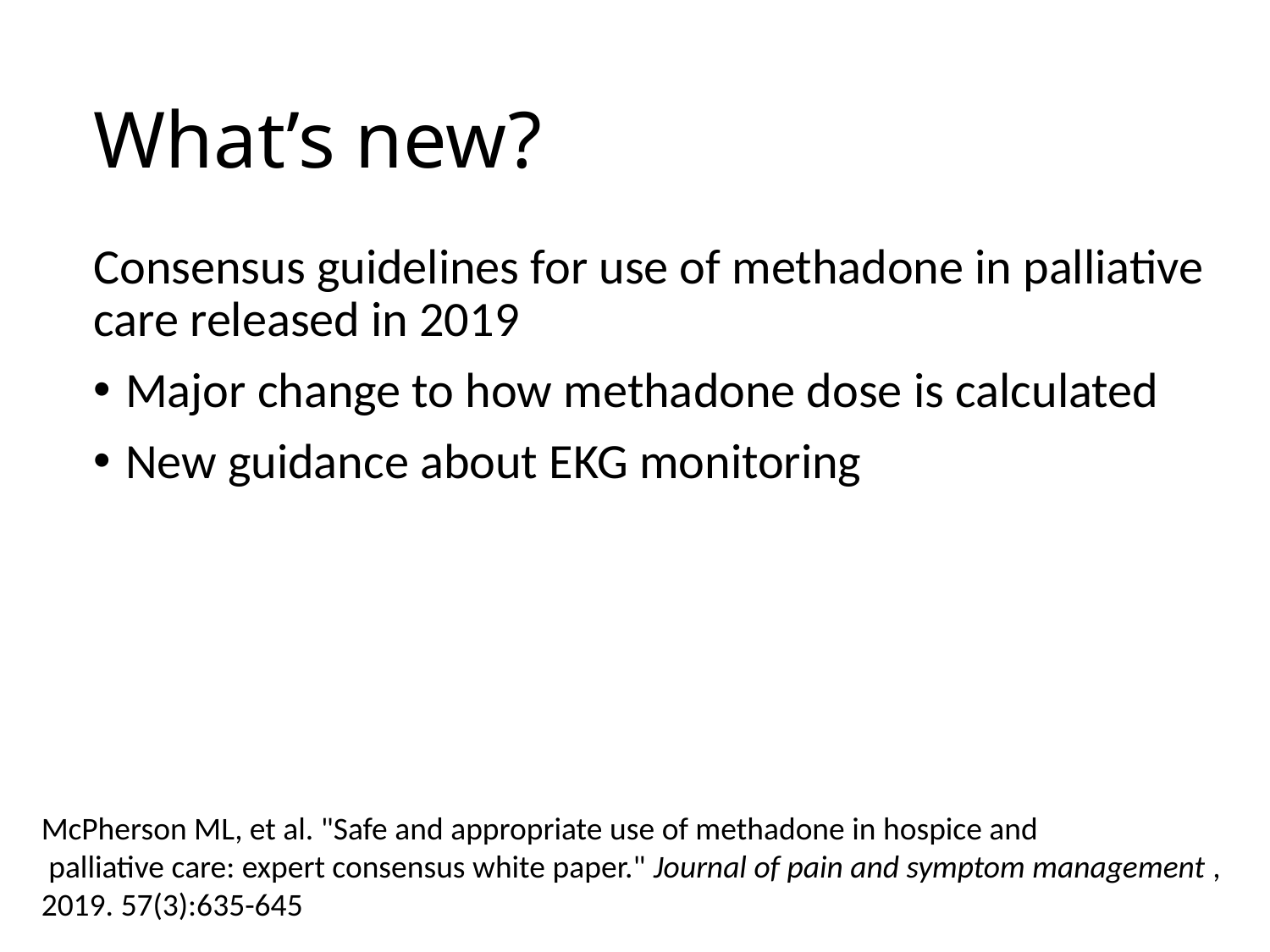

# What’s new?
Consensus guidelines for use of methadone in palliative care released in 2019
Major change to how methadone dose is calculated
New guidance about EKG monitoring
McPherson ML, et al. "Safe and appropriate use of methadone in hospice and
 palliative care: expert consensus white paper." Journal of pain and symptom management ,
2019. 57(3):635-645

## Slide 3
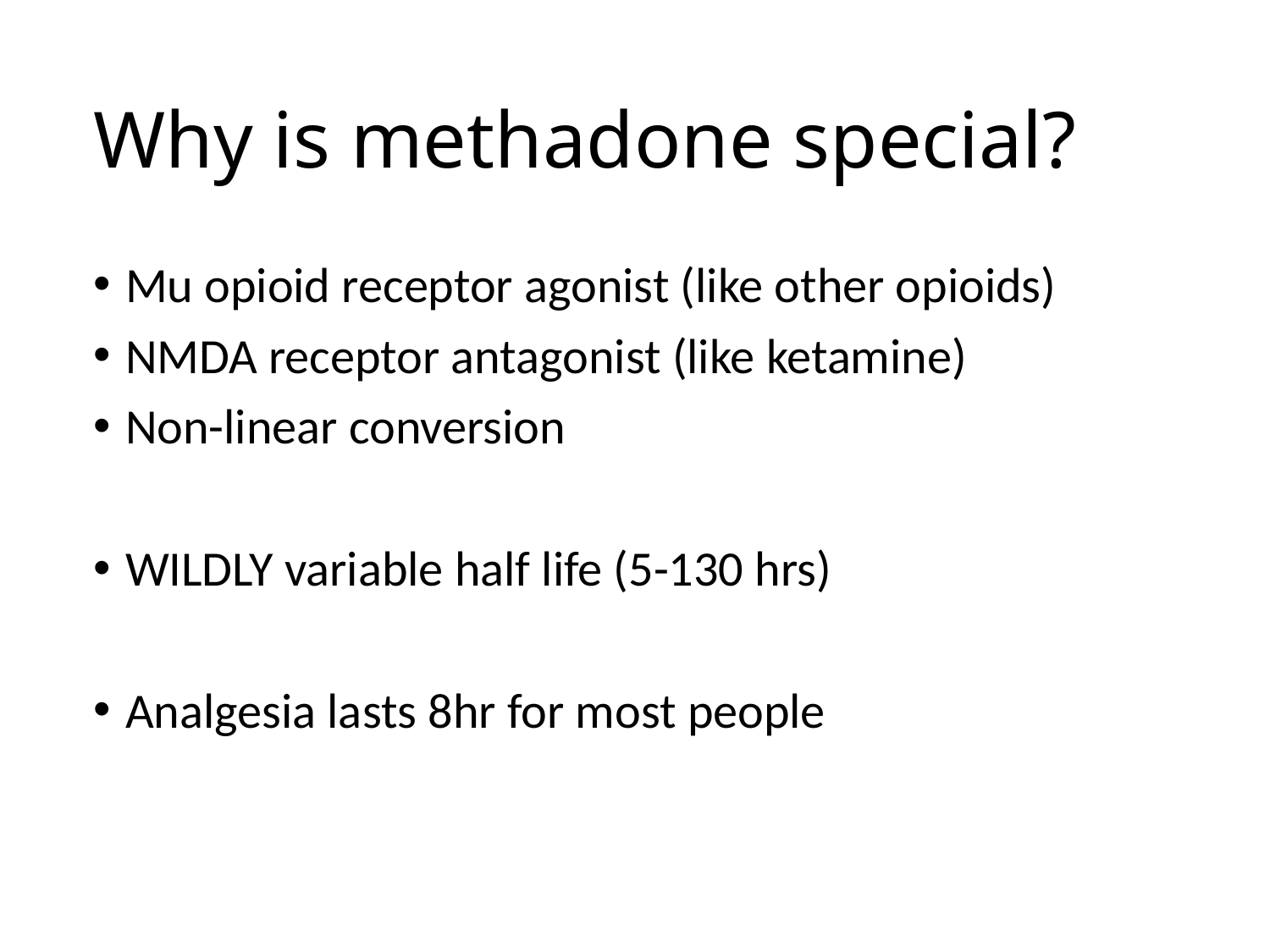

# Why is methadone special?
Mu opioid receptor agonist (like other opioids)
NMDA receptor antagonist (like ketamine)
Non-linear conversion
WILDLY variable half life (5-130 hrs)
Analgesia lasts 8hr for most people

## Slide 4
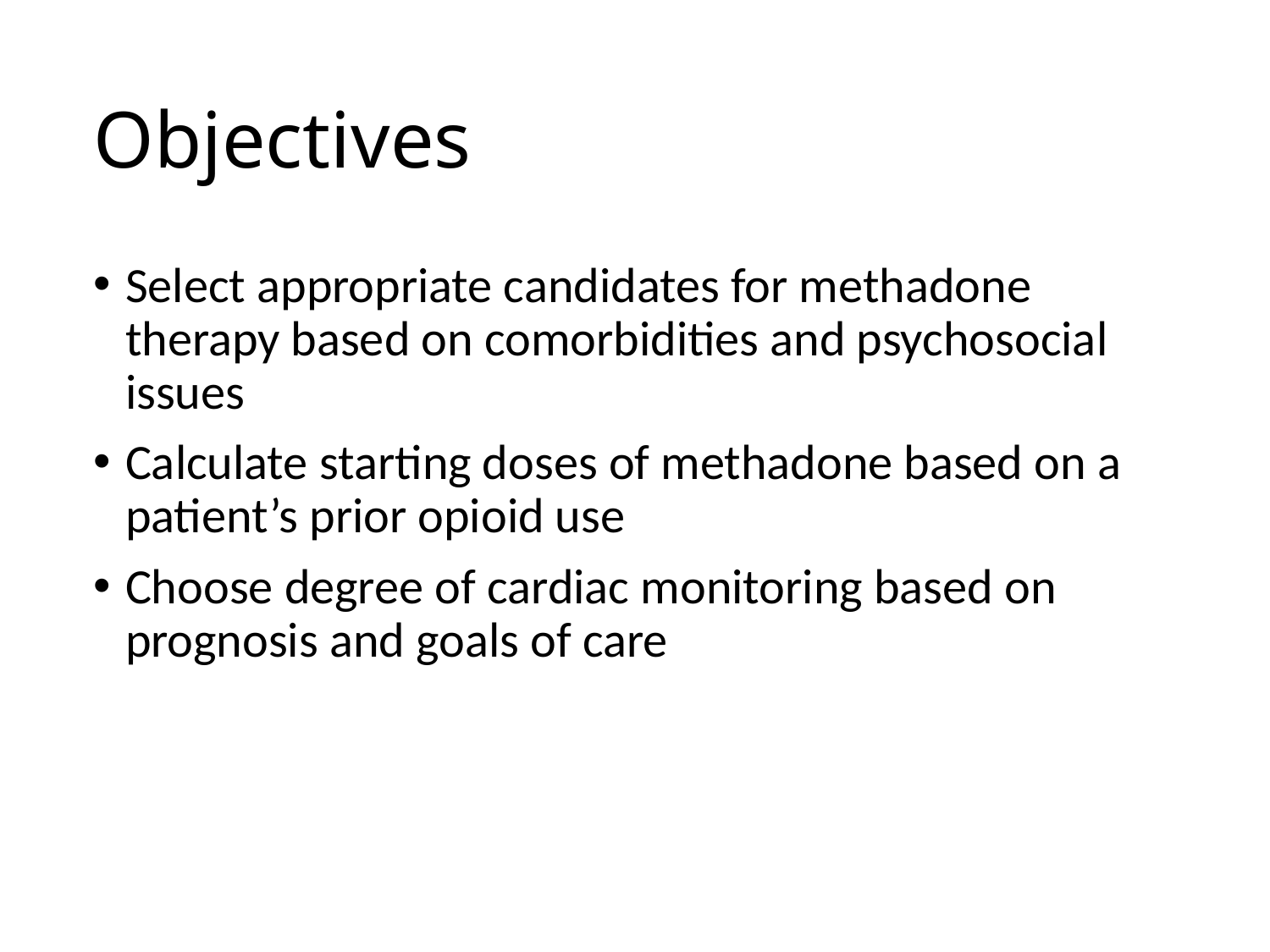

# Objectives
Select appropriate candidates for methadone therapy based on comorbidities and psychosocial issues
Calculate starting doses of methadone based on a patient’s prior opioid use
Choose degree of cardiac monitoring based on prognosis and goals of care

## Slide 5
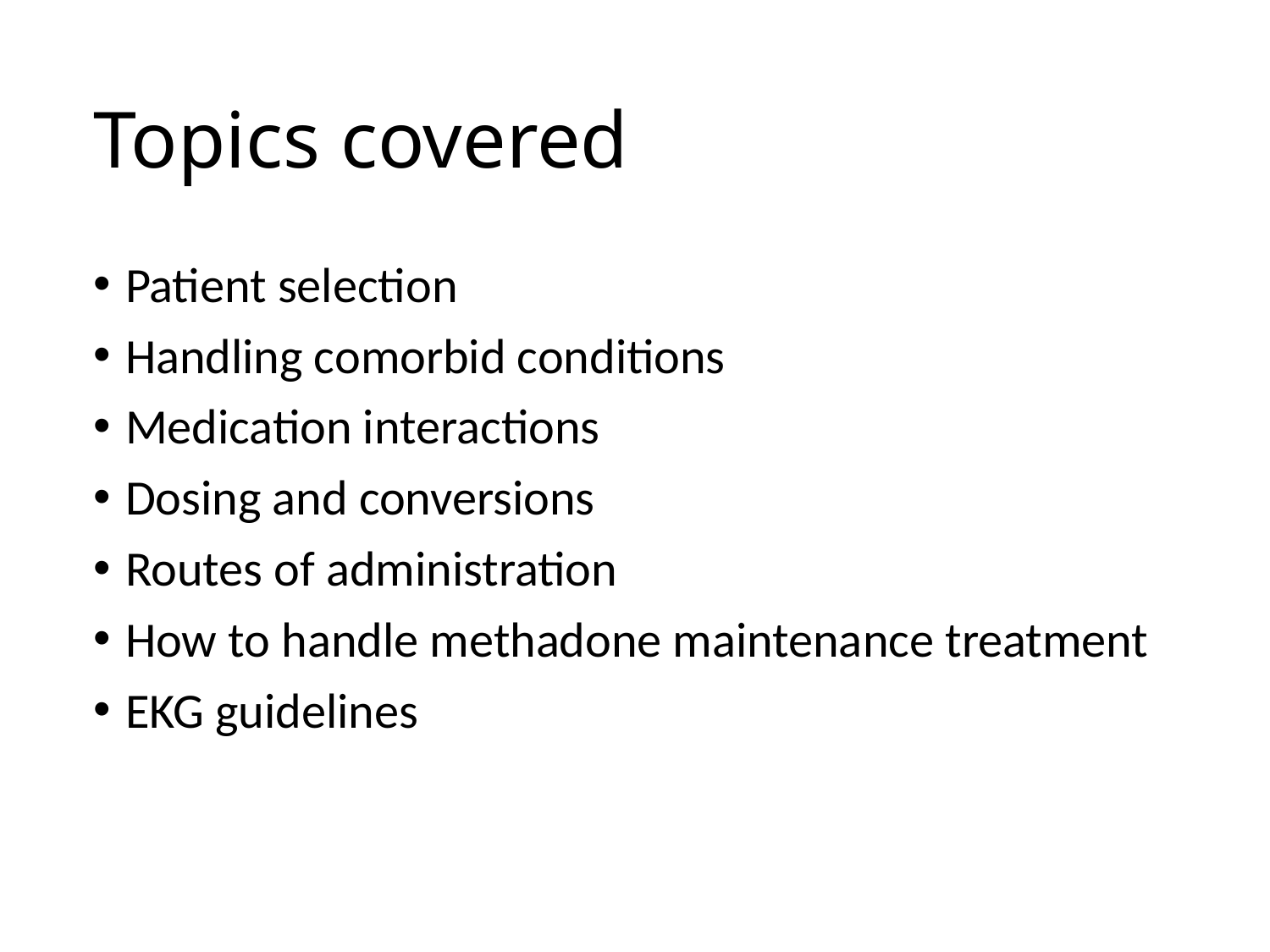

# Topics covered
Patient selection
Handling comorbid conditions
Medication interactions
Dosing and conversions
Routes of administration
How to handle methadone maintenance treatment
EKG guidelines

## Slide 6
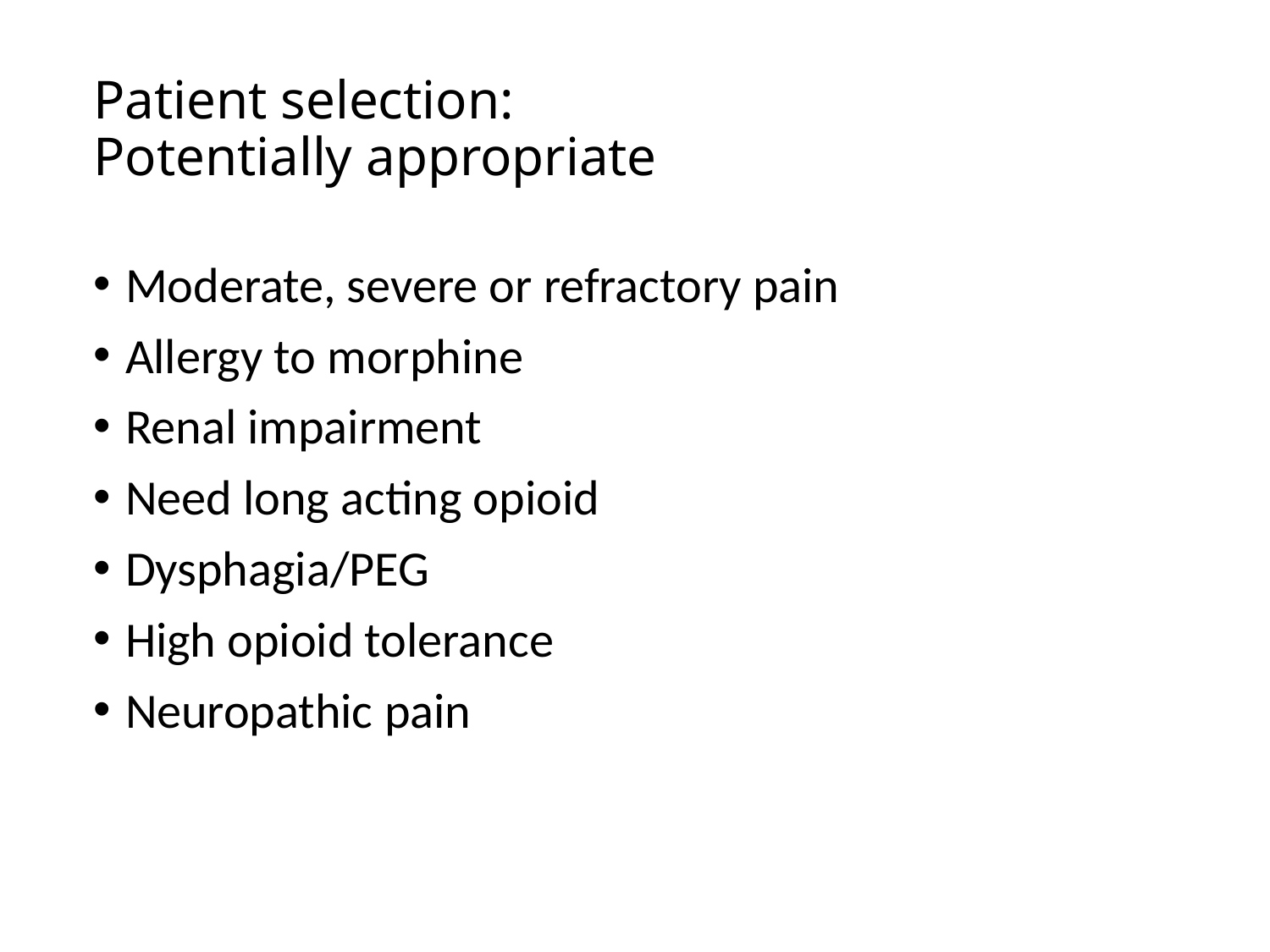

# Patient selection: Potentially appropriate
Moderate, severe or refractory pain
Allergy to morphine
Renal impairment
Need long acting opioid
Dysphagia/PEG
High opioid tolerance
Neuropathic pain

## Slide 7
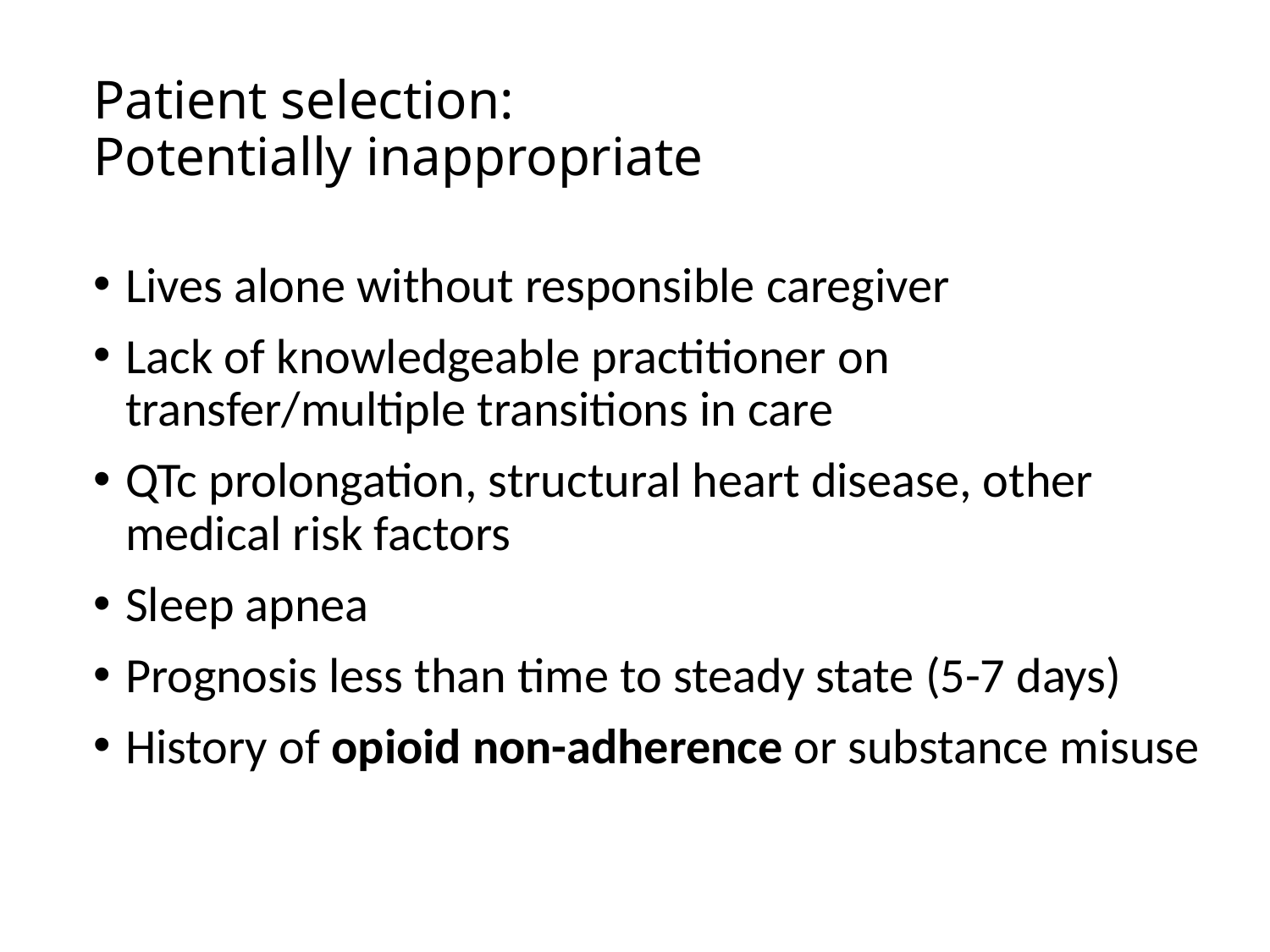

# Patient selection: Potentially inappropriate
Lives alone without responsible caregiver
Lack of knowledgeable practitioner on transfer/multiple transitions in care
QTc prolongation, structural heart disease, other medical risk factors
Sleep apnea
Prognosis less than time to steady state (5-7 days)
History of opioid non-adherence or substance misuse

## Slide 8
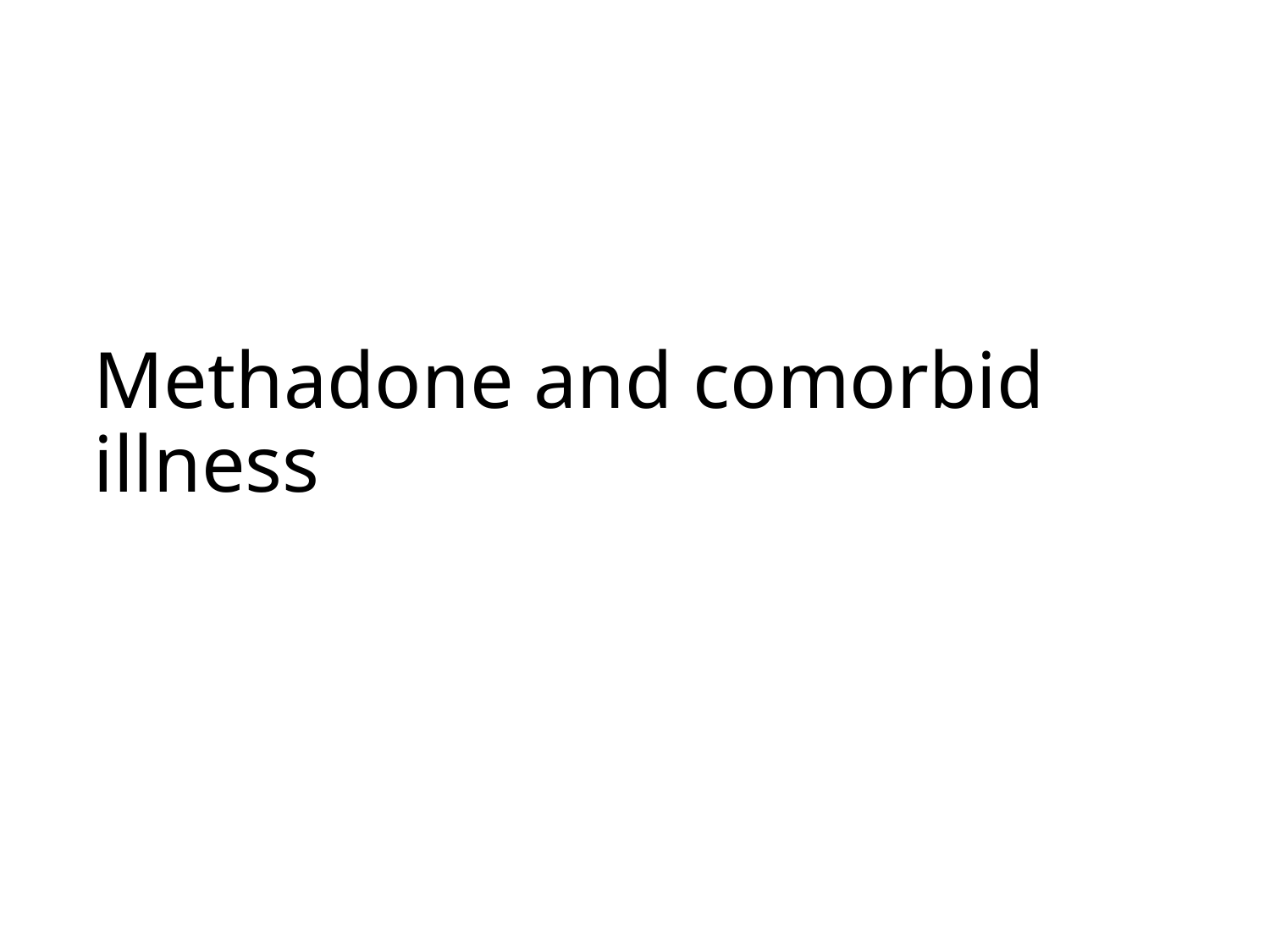

# Methadone and comorbid illness

## Slide 9
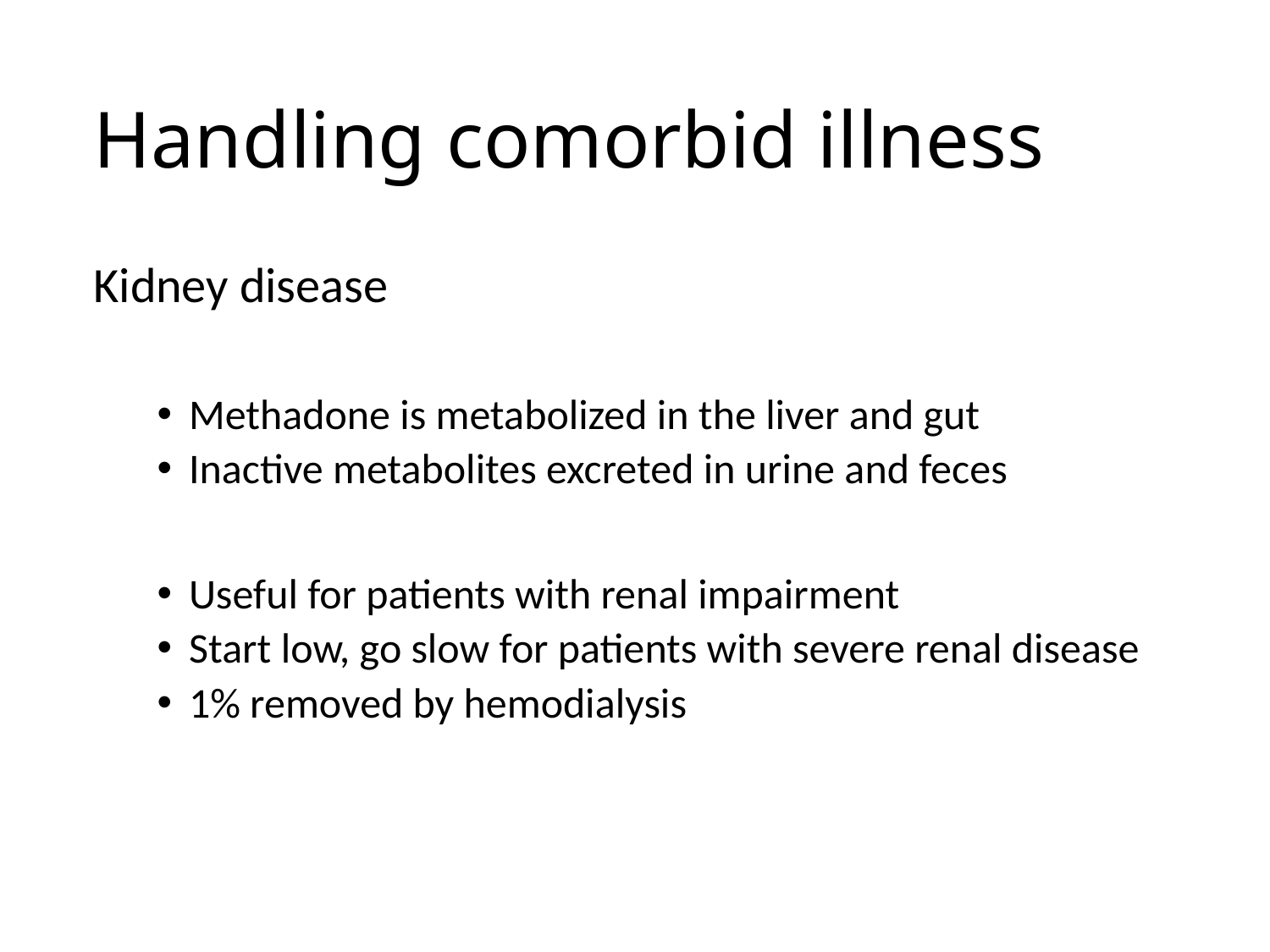

# Handling comorbid illness
Kidney disease
Methadone is metabolized in the liver and gut
Inactive metabolites excreted in urine and feces
Useful for patients with renal impairment
Start low, go slow for patients with severe renal disease
1% removed by hemodialysis

## Slide 10
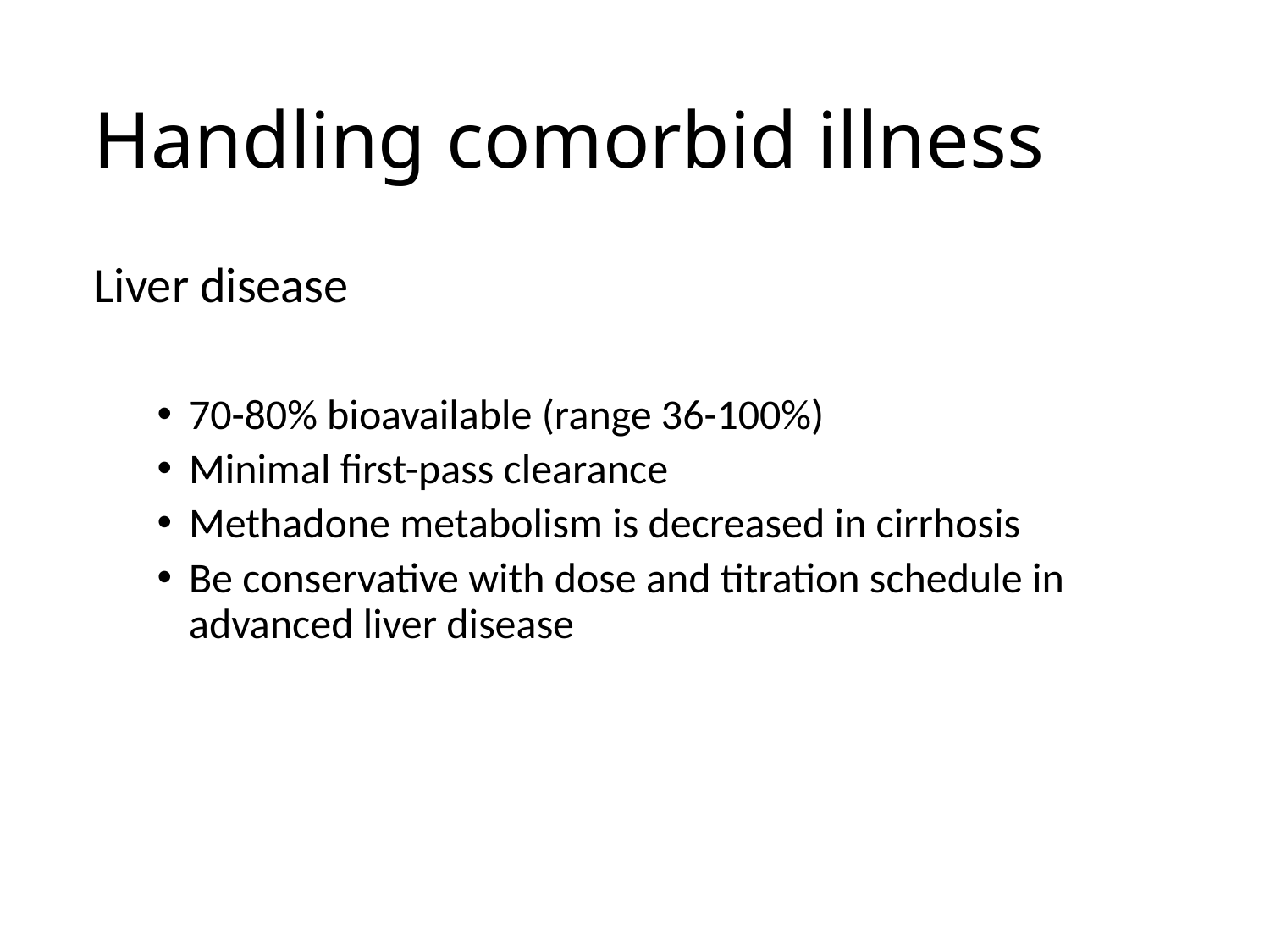

# Handling comorbid illness
Liver disease
70-80% bioavailable (range 36-100%)
Minimal first-pass clearance
Methadone metabolism is decreased in cirrhosis
Be conservative with dose and titration schedule in advanced liver disease

## Slide 11
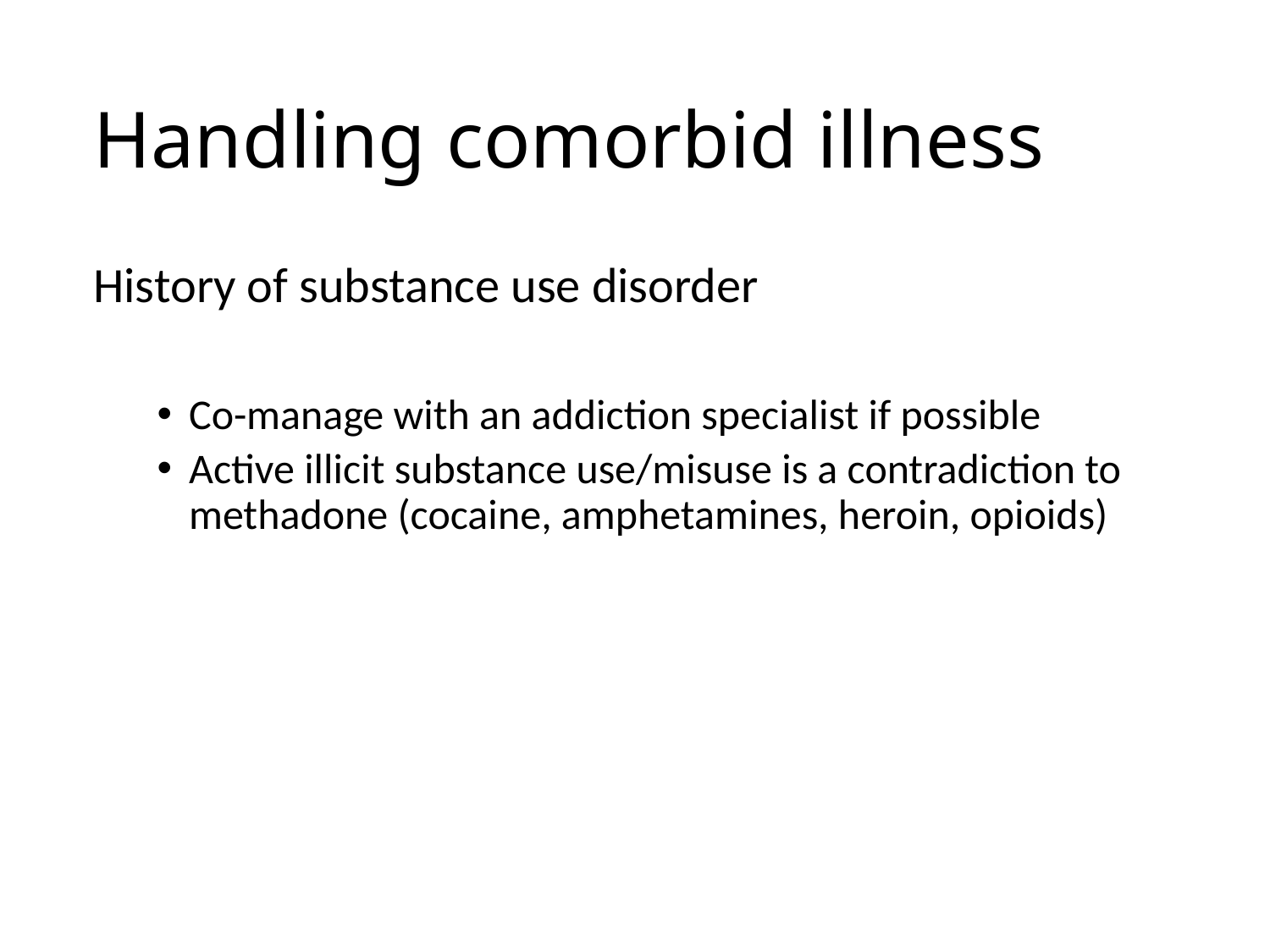

# Handling comorbid illness
History of substance use disorder
Co-manage with an addiction specialist if possible
Active illicit substance use/misuse is a contradiction to methadone (cocaine, amphetamines, heroin, opioids)

## Slide 12
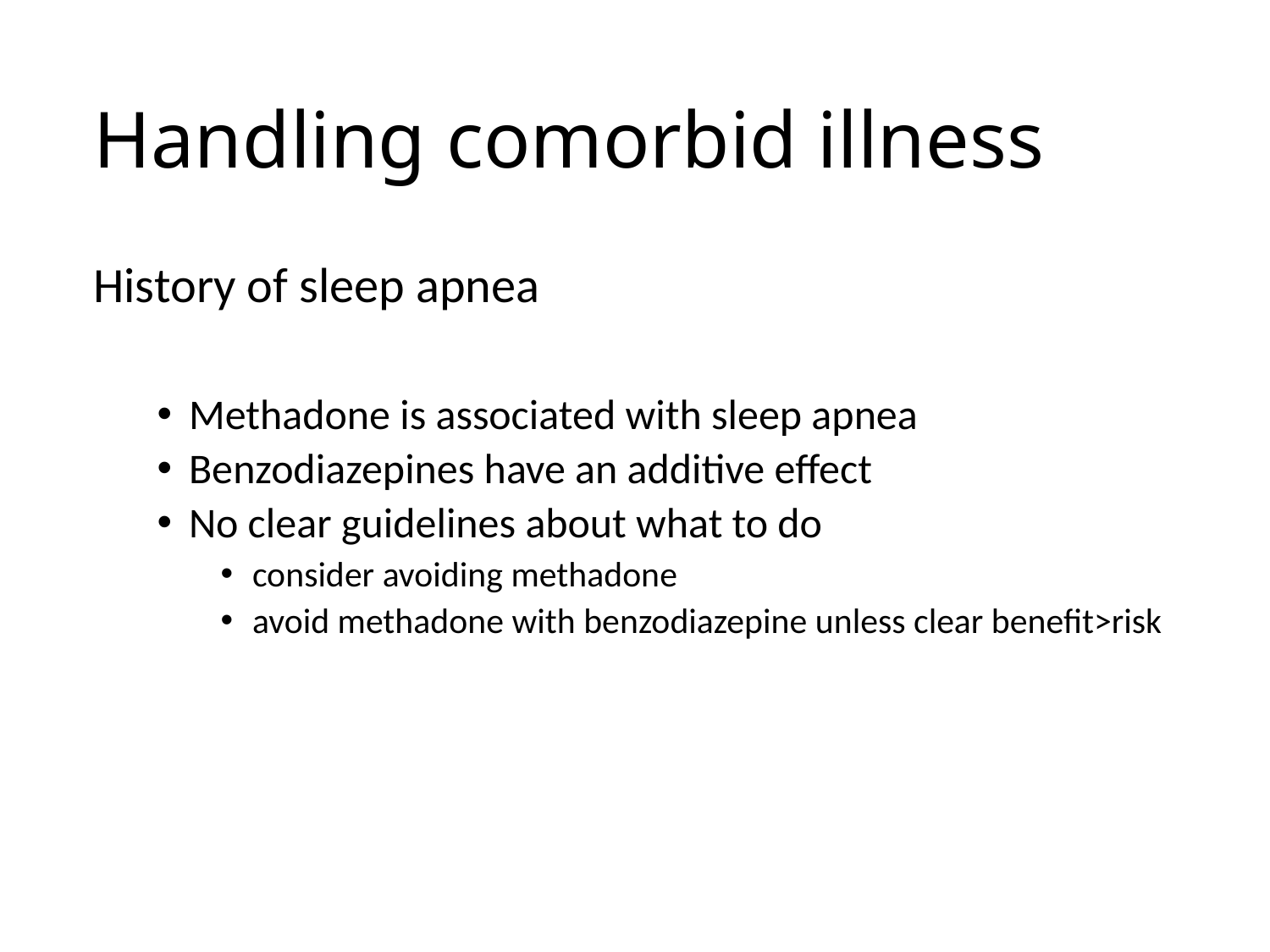

# Handling comorbid illness
History of sleep apnea
Methadone is associated with sleep apnea
Benzodiazepines have an additive effect
No clear guidelines about what to do
consider avoiding methadone
avoid methadone with benzodiazepine unless clear benefit>risk

## Slide 13
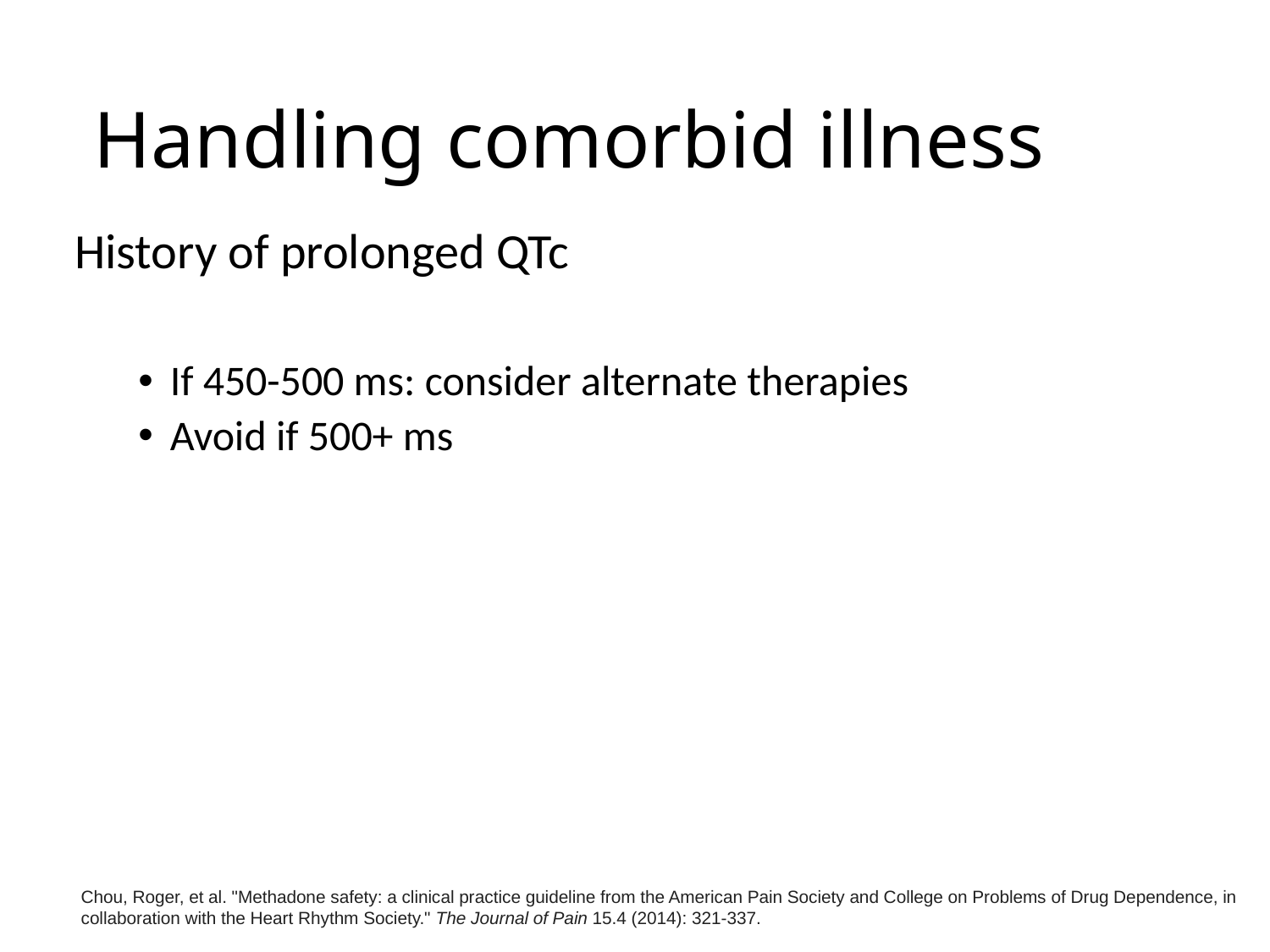

# Handling comorbid illness
History of prolonged QTc
If 450-500 ms: consider alternate therapies
Avoid if 500+ ms
Chou, Roger, et al. "Methadone safety: a clinical practice guideline from the American Pain Society and College on Problems of Drug Dependence, in collaboration with the Heart Rhythm Society." The Journal of Pain 15.4 (2014): 321-337.

## Slide 14
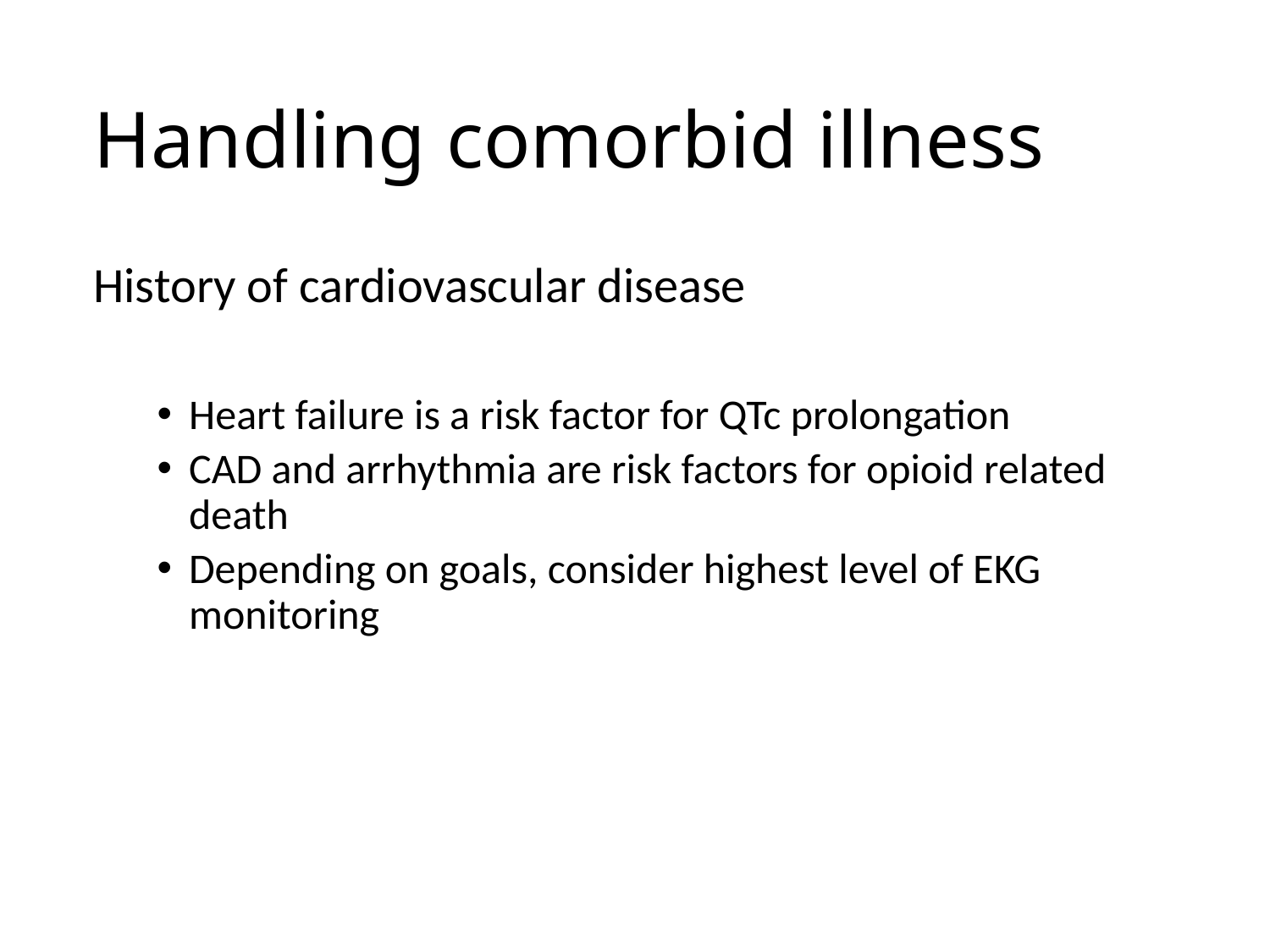

# Handling comorbid illness
History of cardiovascular disease
Heart failure is a risk factor for QTc prolongation
CAD and arrhythmia are risk factors for opioid related death
Depending on goals, consider highest level of EKG monitoring

## Slide 15
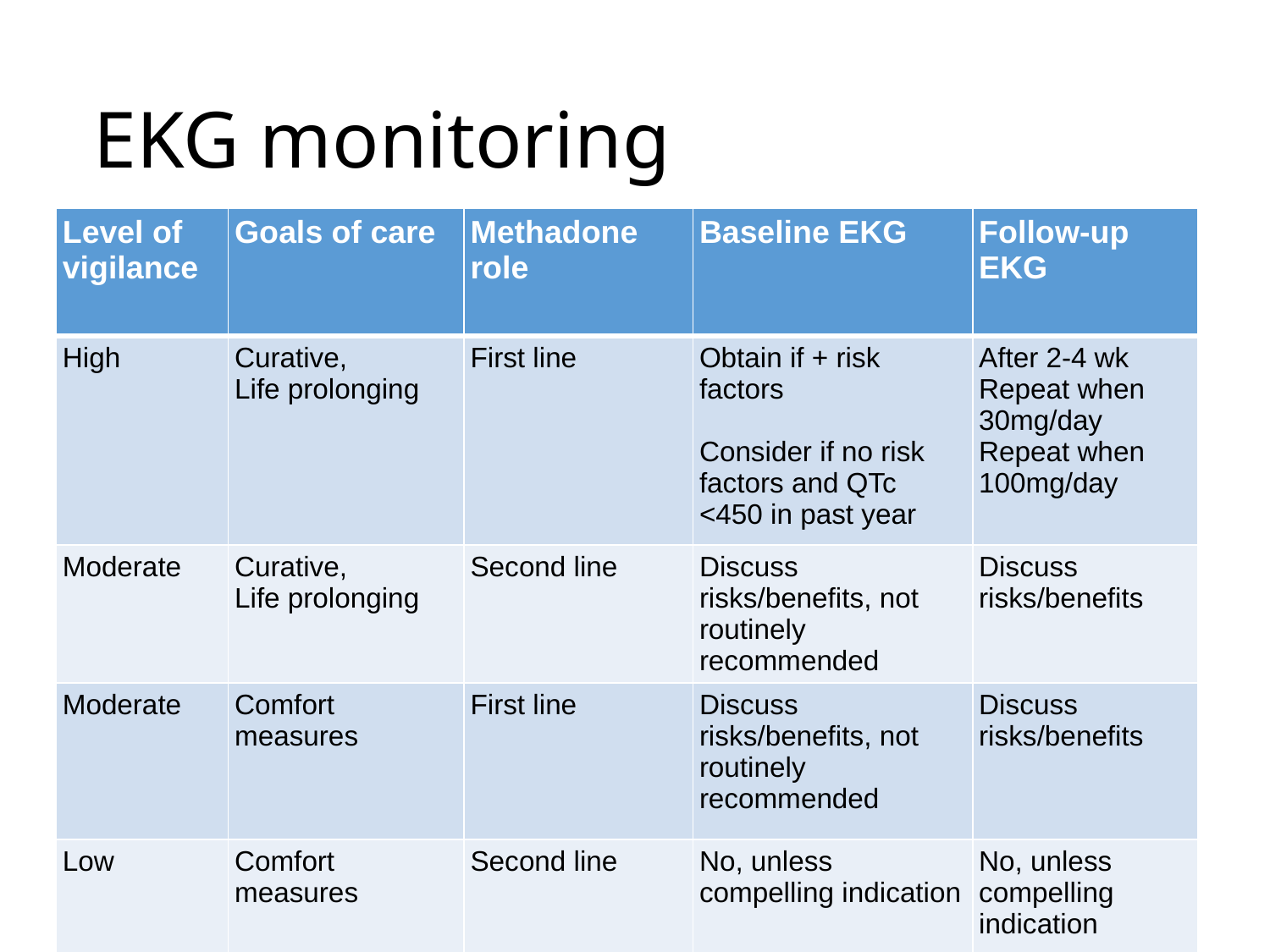

# EKG monitoring
| Level of vigilance | Goals of care | Methadone role | Baseline EKG | Follow-up EKG |
| --- | --- | --- | --- | --- |
| High | Curative, Life prolonging | First line | Obtain if + risk factors Consider if no risk factors and QTc <450 in past year | After 2-4 wk Repeat when 30mg/day Repeat when 100mg/day |
| Moderate | Curative, Life prolonging | Second line | Discuss risks/benefits, not routinely recommended | Discuss risks/benefits |
| Moderate | Comfort measures | First line | Discuss risks/benefits, not routinely recommended | Discuss risks/benefits |
| Low | Comfort measures | Second line | No, unless compelling indication | No, unless compelling indication |

## Slide 16
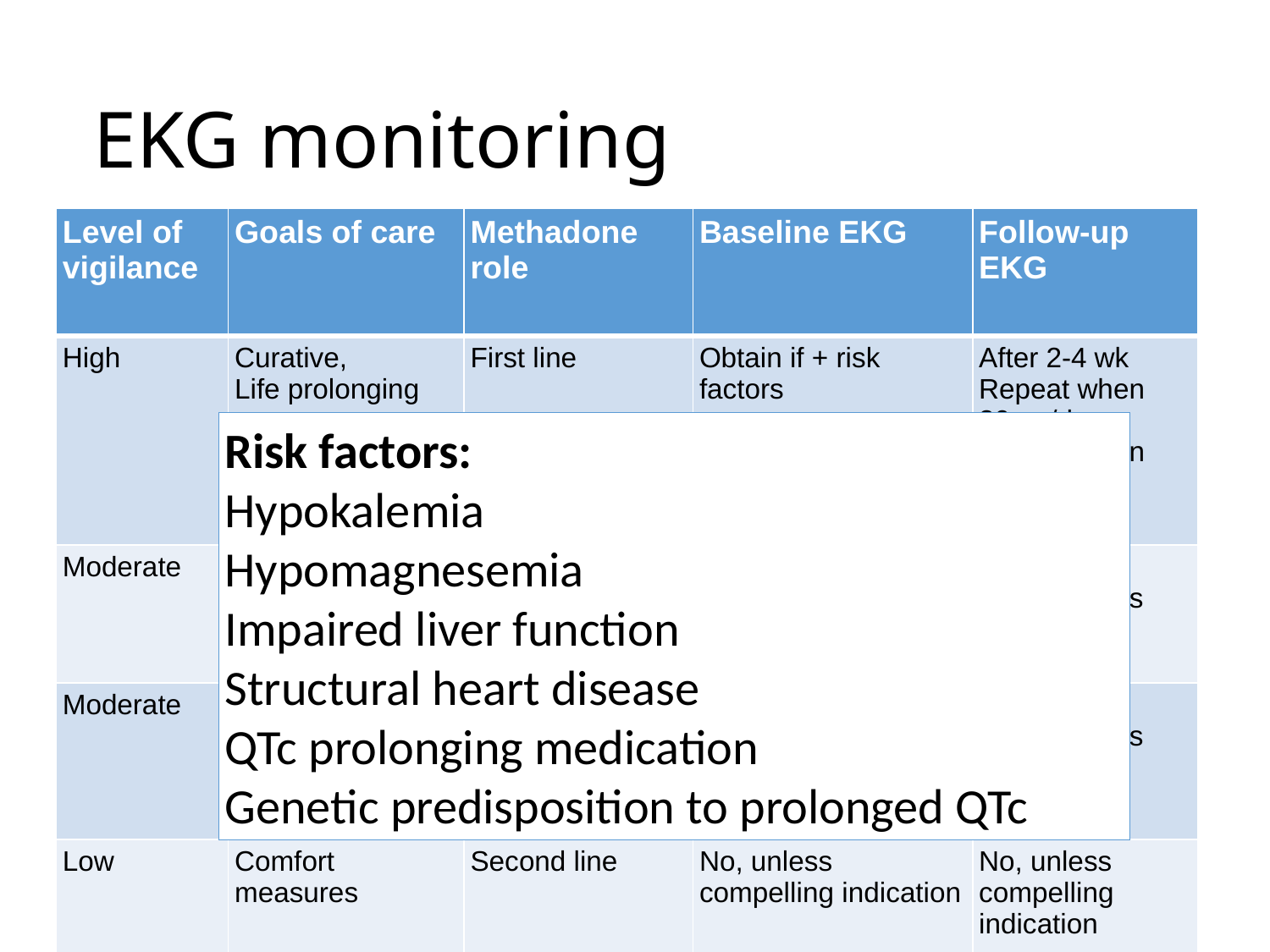

# EKG monitoring
| Level of vigilance | Goals of care | Methadone role | Baseline EKG | Follow-up EKG |
| --- | --- | --- | --- | --- |
| High | Curative, Life prolonging | First line | Obtain if + risk factors Consider if no risk factors and QTc <450 in past year | After 2-4 wk Repeat when 30mg/day Repeat when 100mg/day |
| Moderate | Curative, Life prolonging | Second line | Discuss risks/benefits, not routinely recommended | Discuss risks/benefits |
| Moderate | Comfort measures | First line | Discuss risks/benefits, not routinely recommended | Discuss risks/benefits |
| Low | Comfort measures | Second line | No, unless compelling indication | No, unless compelling indication |
Risk factors:
Hypokalemia
Hypomagnesemia
Impaired liver function
Structural heart disease
QTc prolonging medication
Genetic predisposition to prolonged QTc

## Slide 17
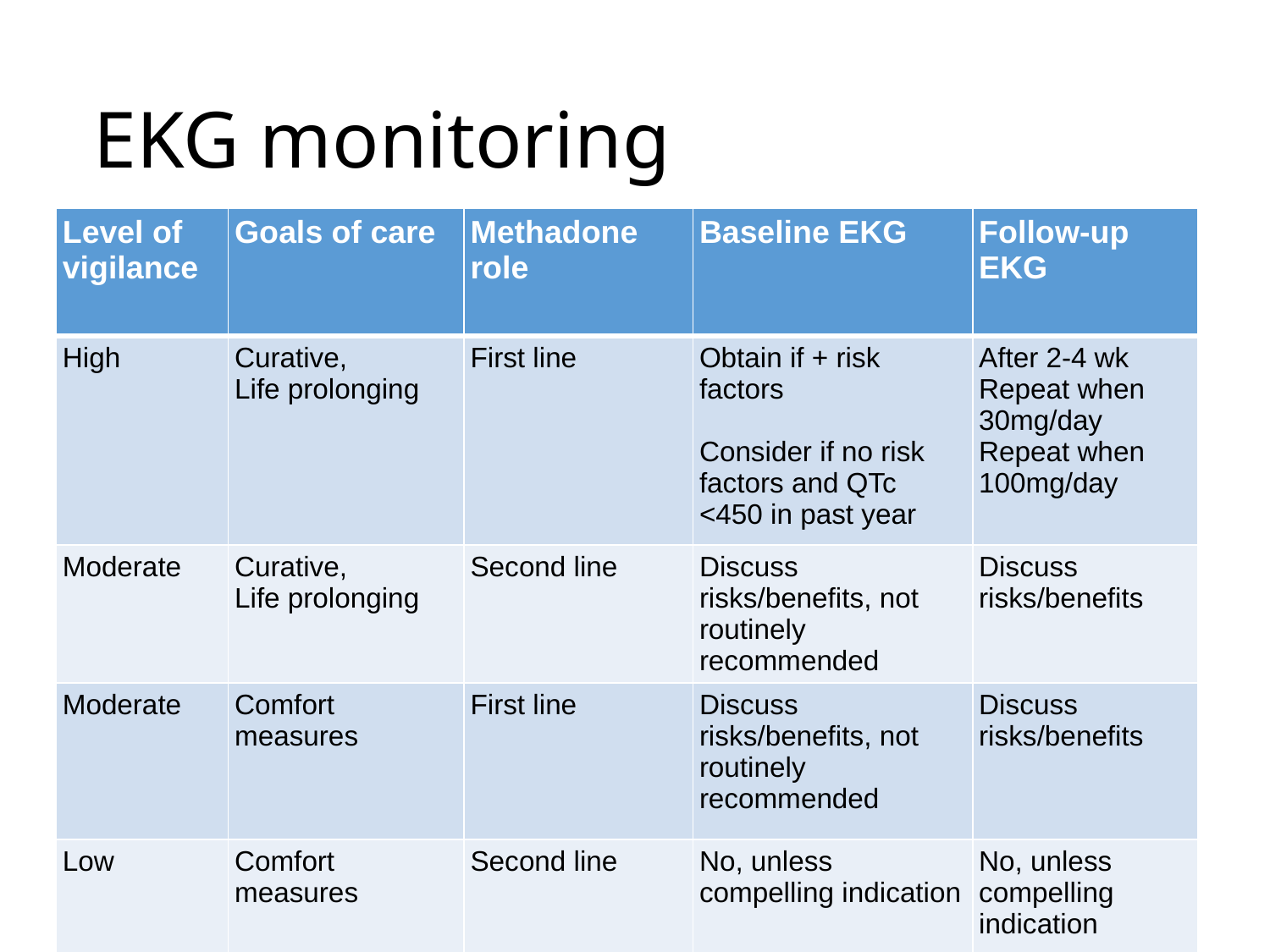

# EKG monitoring
| Level of vigilance | Goals of care | Methadone role | Baseline EKG | Follow-up EKG |
| --- | --- | --- | --- | --- |
| High | Curative, Life prolonging | First line | Obtain if + risk factors Consider if no risk factors and QTc <450 in past year | After 2-4 wk Repeat when 30mg/day Repeat when 100mg/day |
| Moderate | Curative, Life prolonging | Second line | Discuss risks/benefits, not routinely recommended | Discuss risks/benefits |
| Moderate | Comfort measures | First line | Discuss risks/benefits, not routinely recommended | Discuss risks/benefits |
| Low | Comfort measures | Second line | No, unless compelling indication | No, unless compelling indication |

## Slide 18
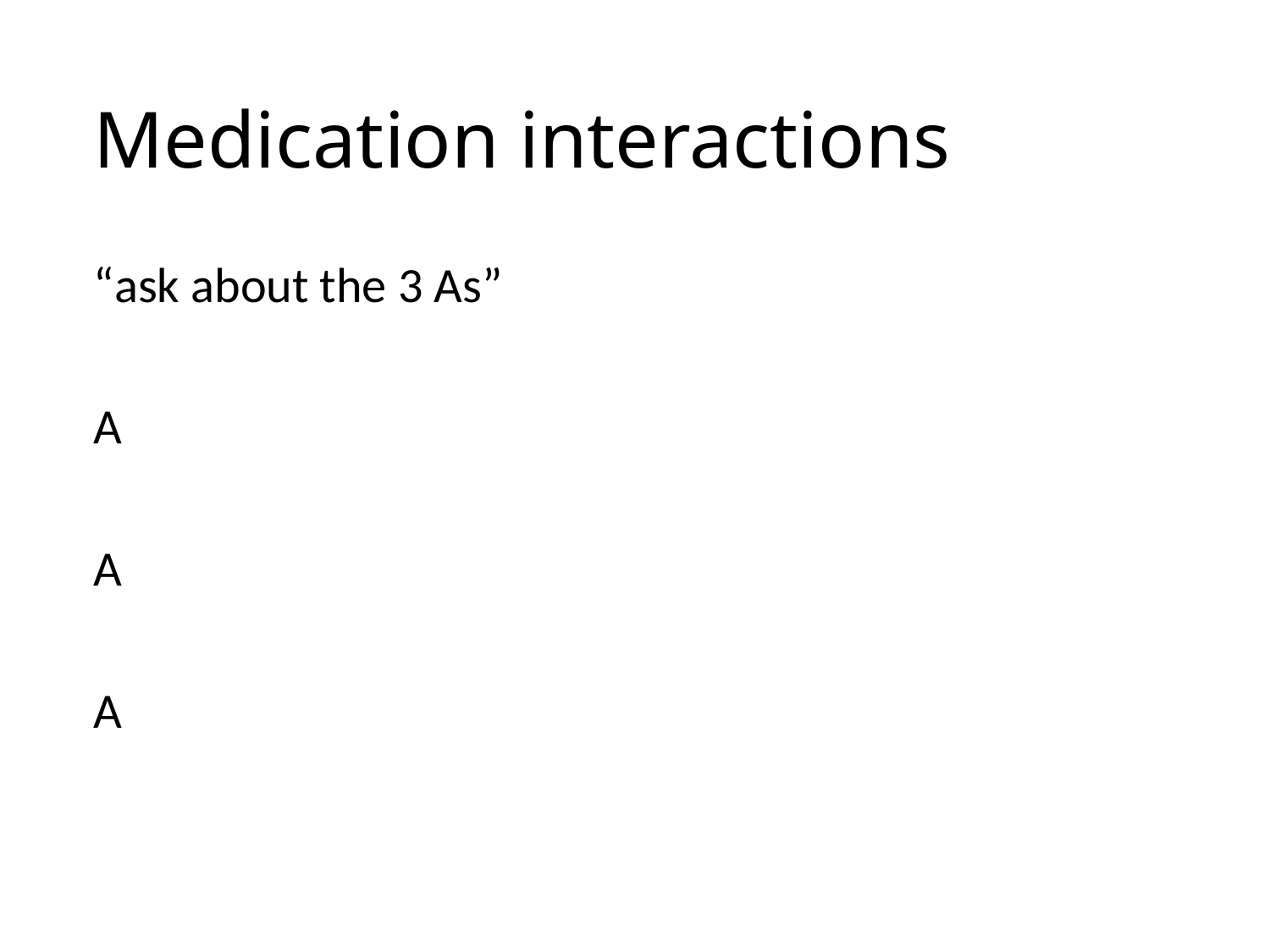

# Medication interactions
“ask about the 3 As”
A
A
A

## Slide 19
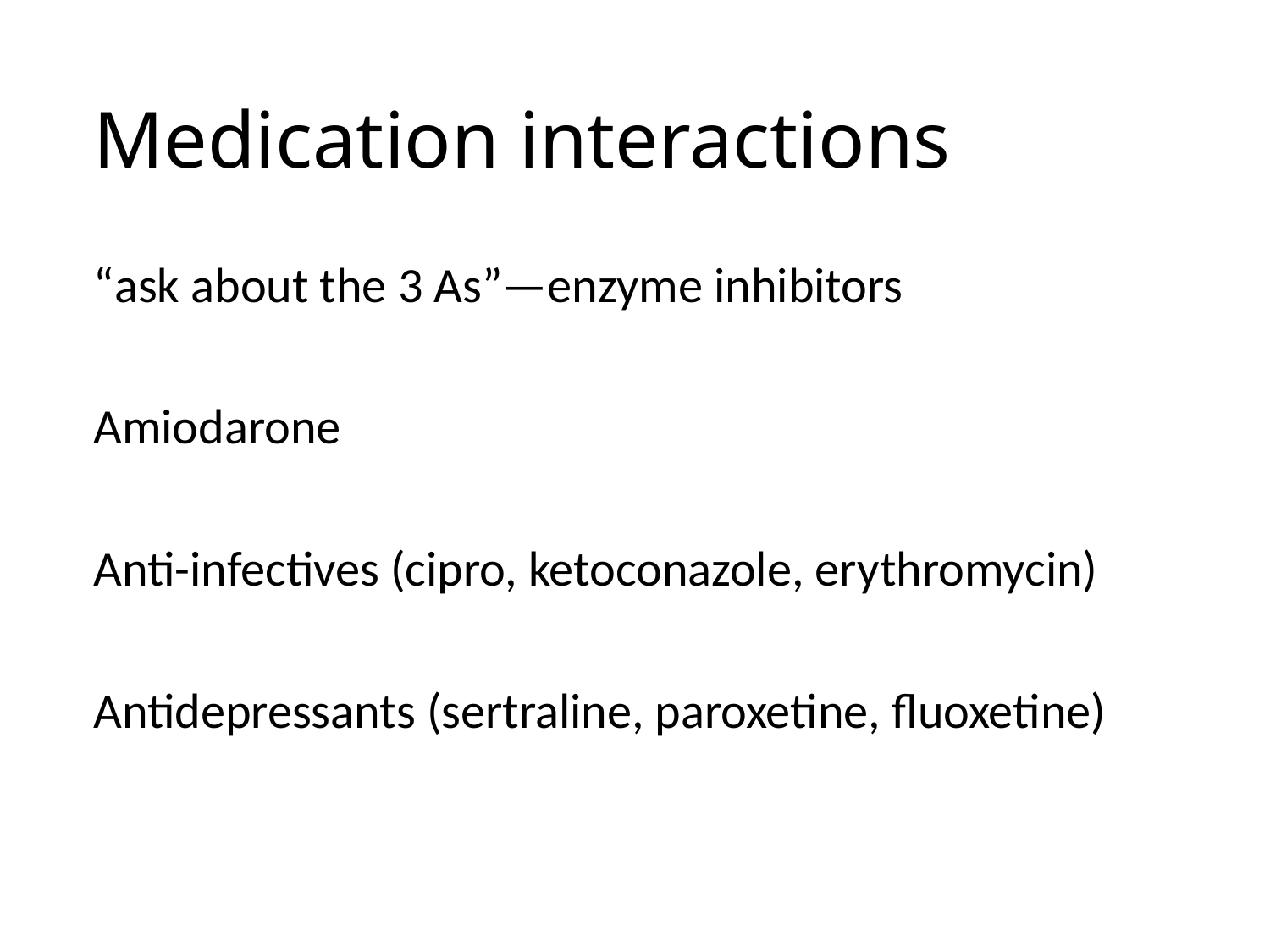

# Medication interactions
“ask about the 3 As”—enzyme inhibitors
Amiodarone
Anti-infectives (cipro, ketoconazole, erythromycin)
Antidepressants (sertraline, paroxetine, fluoxetine)

## Slide 20
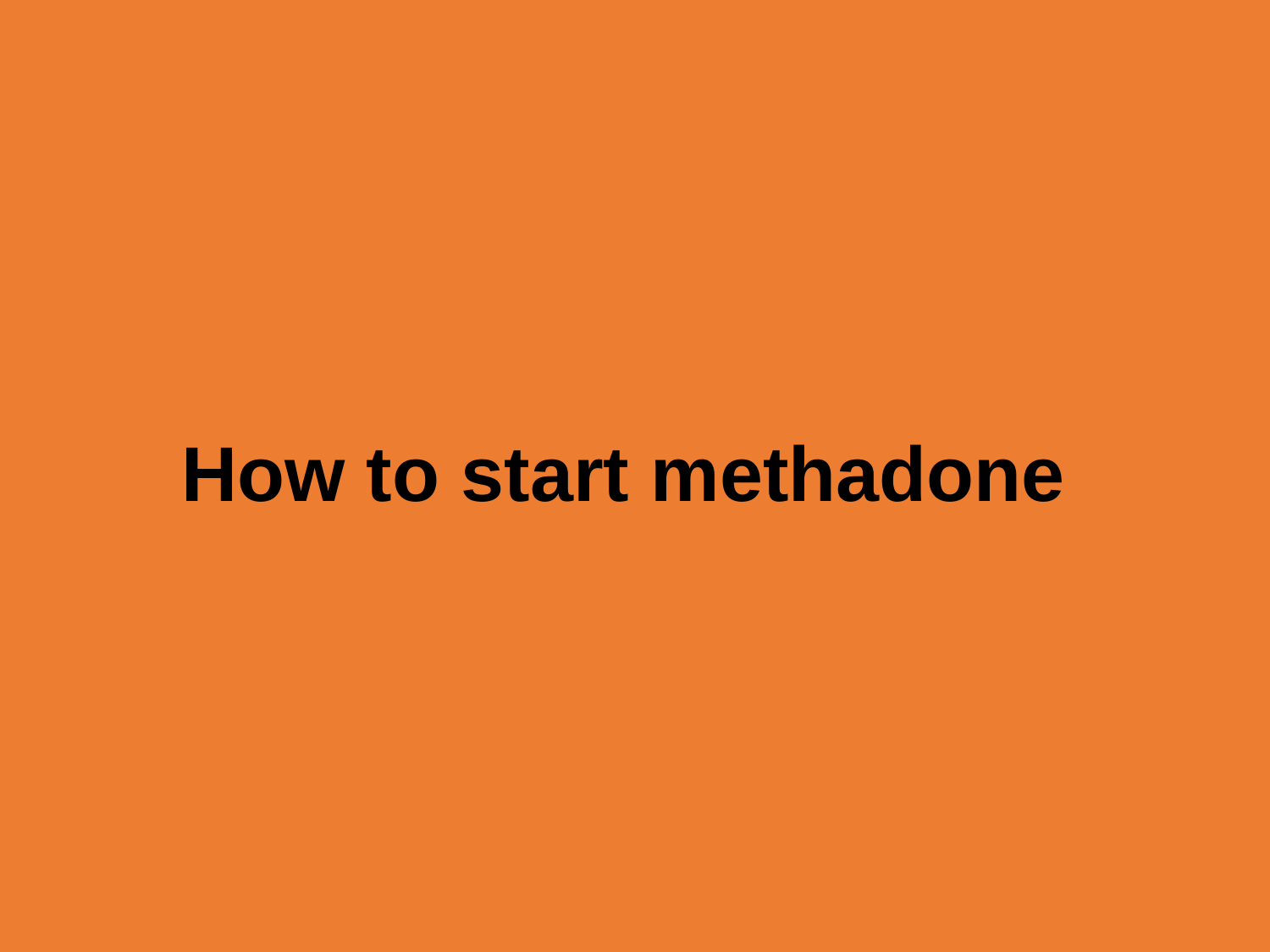

# How to start methadone

## Slide 21
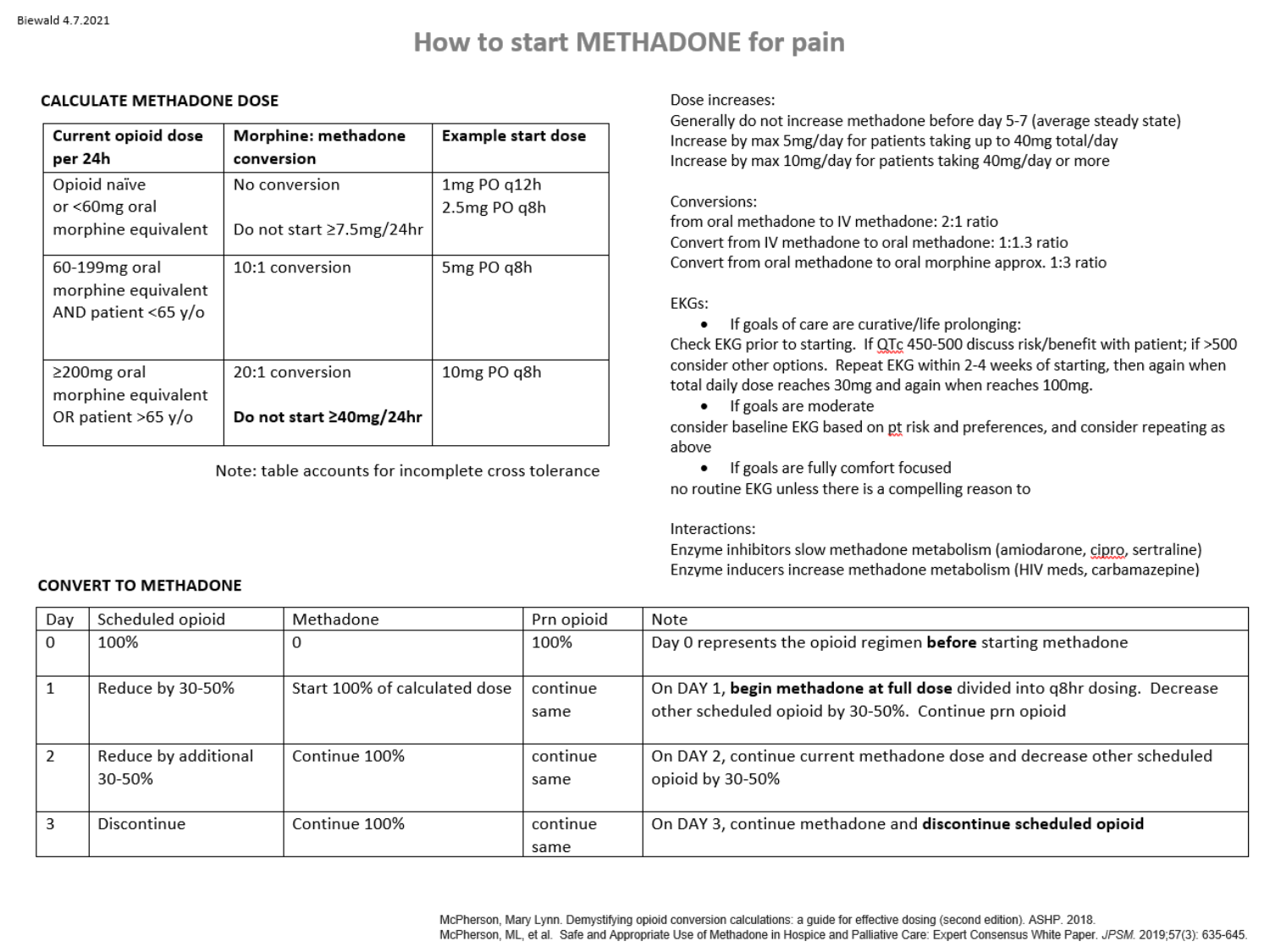

#

## Slide 22
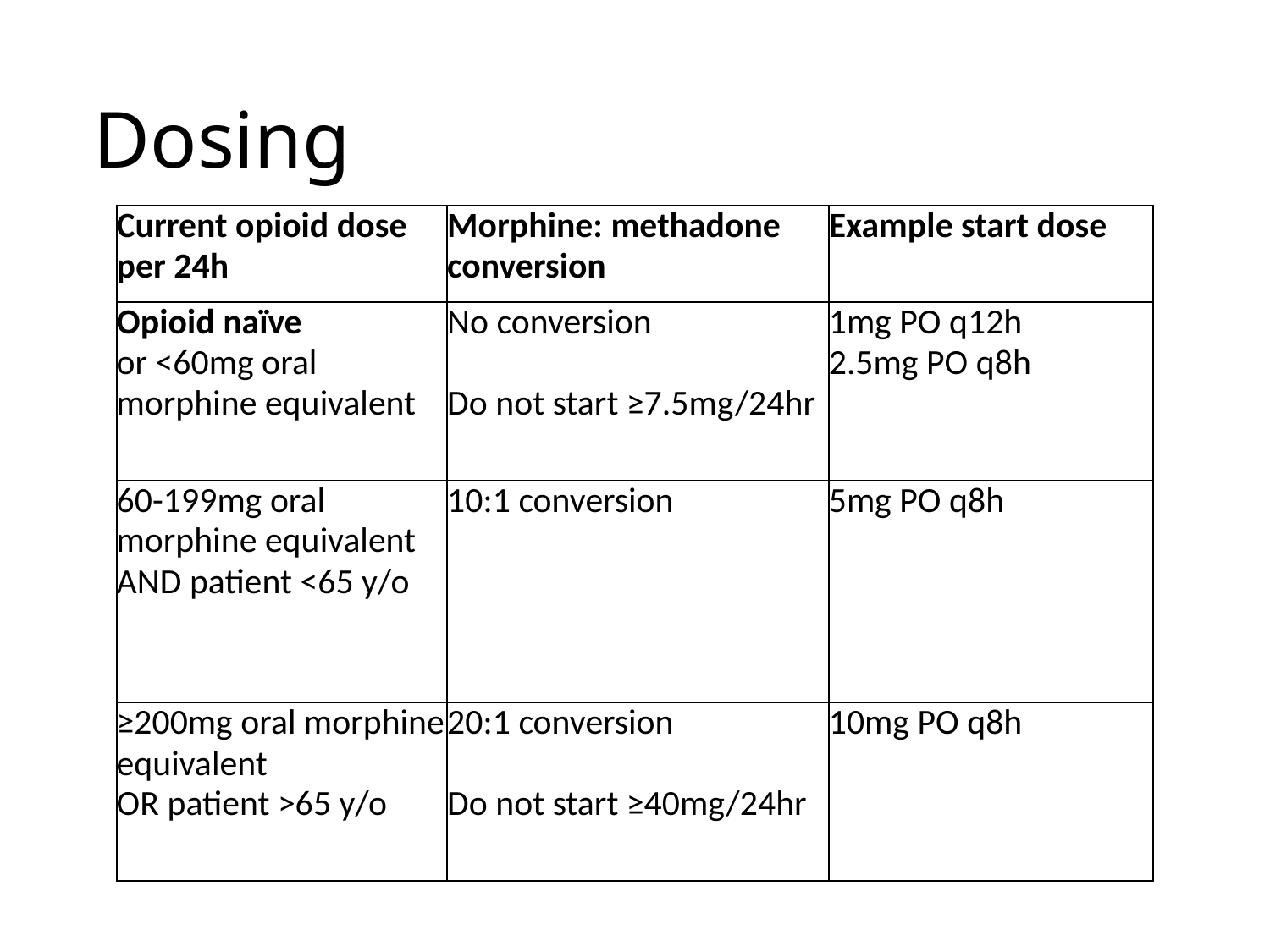

# Dosing
| Current opioid dose per 24h | Morphine: methadone conversion | Example start dose |
| --- | --- | --- |
| Opioid naïve  or <60mg oral morphine equivalent | No conversion Do not start ≥7.5mg/24hr | 1mg PO q12h  2.5mg PO q8h |
| 60-199mg oral morphine equivalent AND patient <65 y/o | 10:1 conversion | 5mg PO q8h |
| ≥200mg oral morphine equivalent OR patient >65 y/o | 20:1 conversion Do not start ≥40mg/24hr | 10mg PO q8h |

## Slide 23
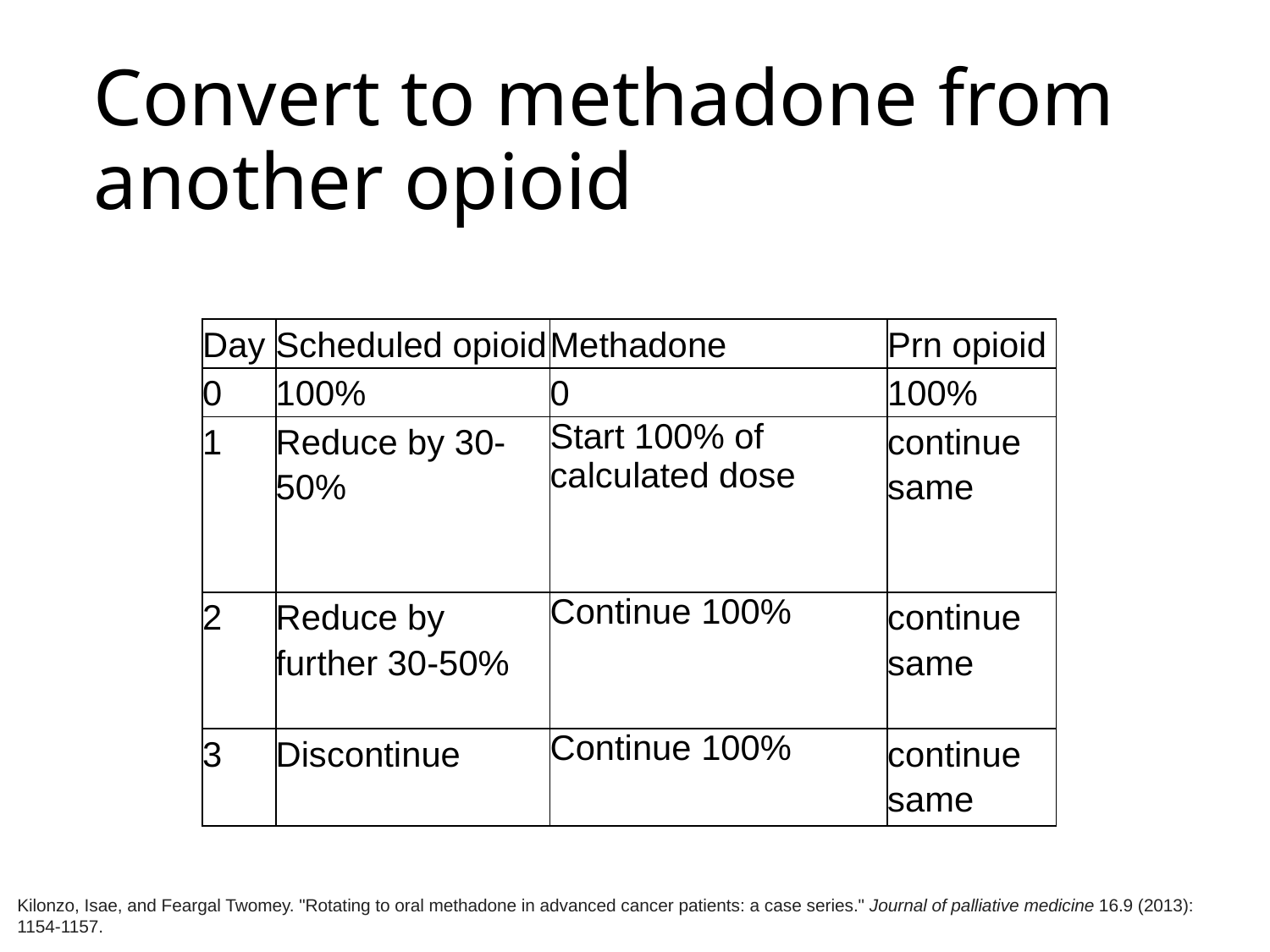

# Convert to methadone from another opioid
| Day | Scheduled opioid | Methadone | Prn opioid |
| --- | --- | --- | --- |
| 0 | 100% | 0 | 100% |
| 1 | Reduce by 30-50% | Start 100% of calculated dose | continue same |
| 2 | Reduce by further 30-50% | Continue 100% | continue same |
| 3 | Discontinue | Continue 100% | continue same |
Kilonzo, Isae, and Feargal Twomey. "Rotating to oral methadone in advanced cancer patients: a case series." Journal of palliative medicine 16.9 (2013): 1154-1157.

## Slide 24
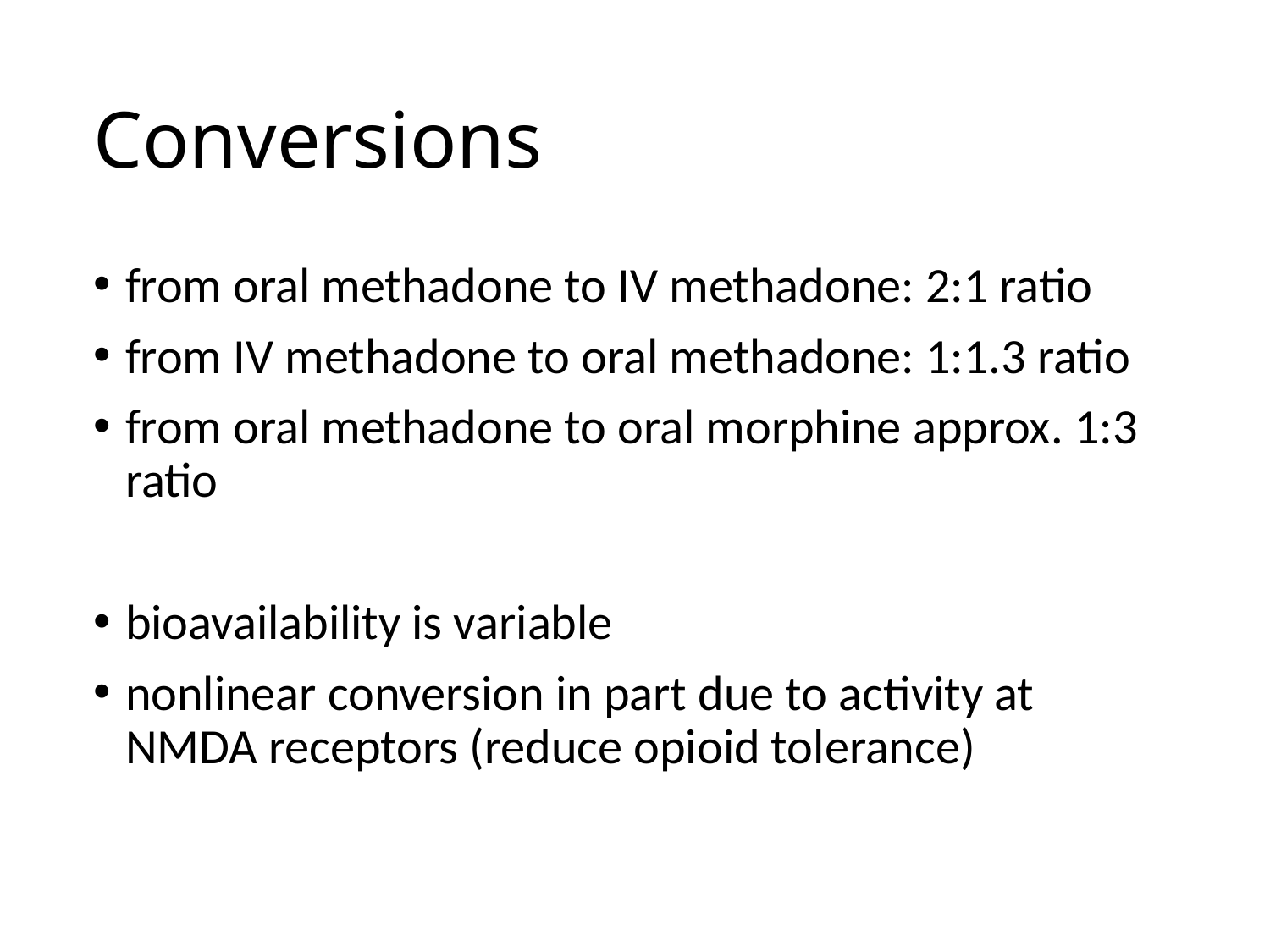

# Conversions
from oral methadone to IV methadone: 2:1 ratio
from IV methadone to oral methadone: 1:1.3 ratio
from oral methadone to oral morphine approx. 1:3 ratio
bioavailability is variable
nonlinear conversion in part due to activity at NMDA receptors (reduce opioid tolerance)

## Slide 25
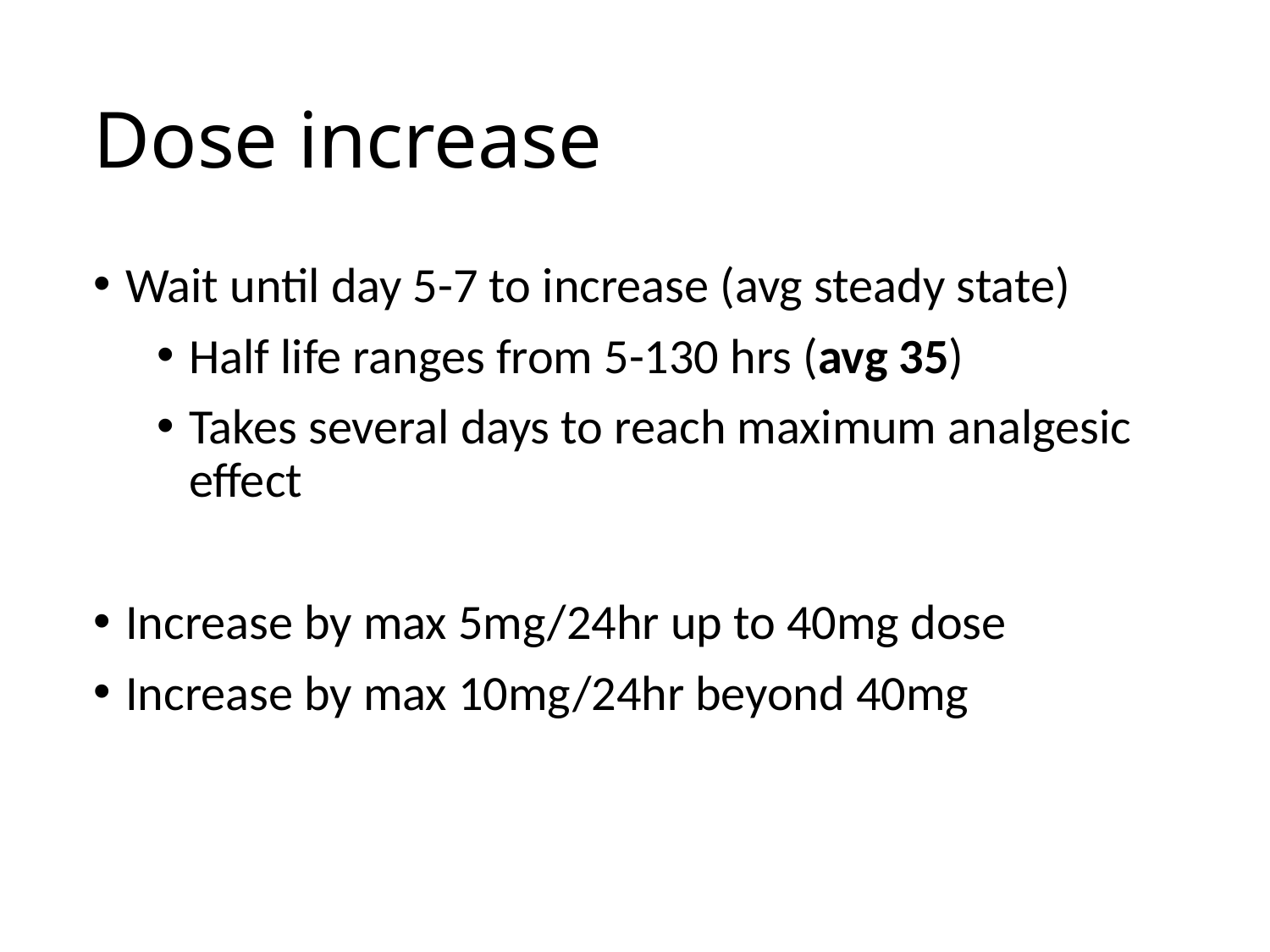

# Dose increase
Wait until day 5-7 to increase (avg steady state)
Half life ranges from 5-130 hrs (avg 35)
Takes several days to reach maximum analgesic effect
Increase by max 5mg/24hr up to 40mg dose
Increase by max 10mg/24hr beyond 40mg

## Slide 26
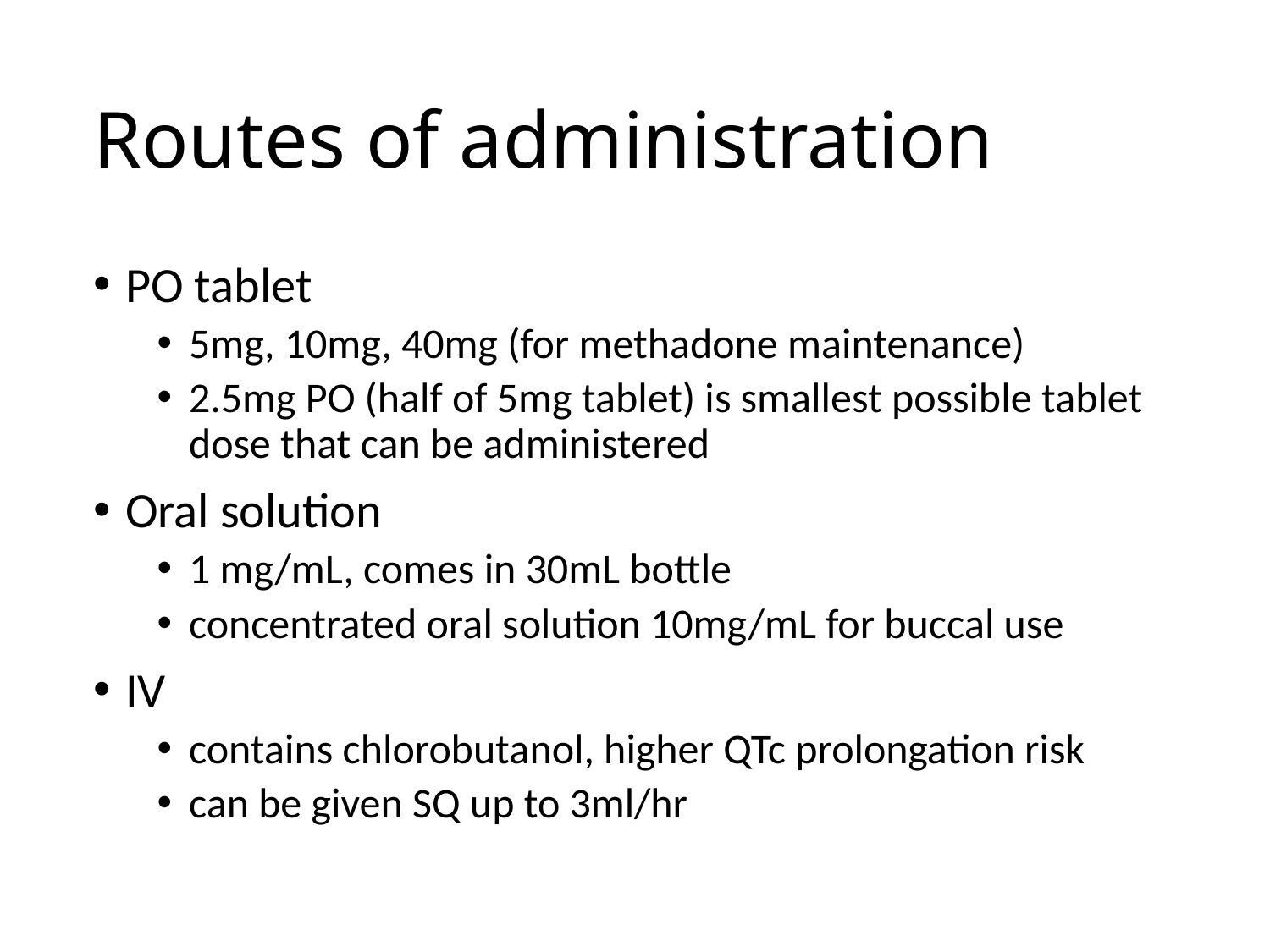

# Routes of administration
PO tablet
5mg, 10mg, 40mg (for methadone maintenance)
2.5mg PO (half of 5mg tablet) is smallest possible tablet dose that can be administered
Oral solution
1 mg/mL, comes in 30mL bottle
concentrated oral solution 10mg/mL for buccal use
IV
contains chlorobutanol, higher QTc prolongation risk
can be given SQ up to 3ml/hr

## Slide 27
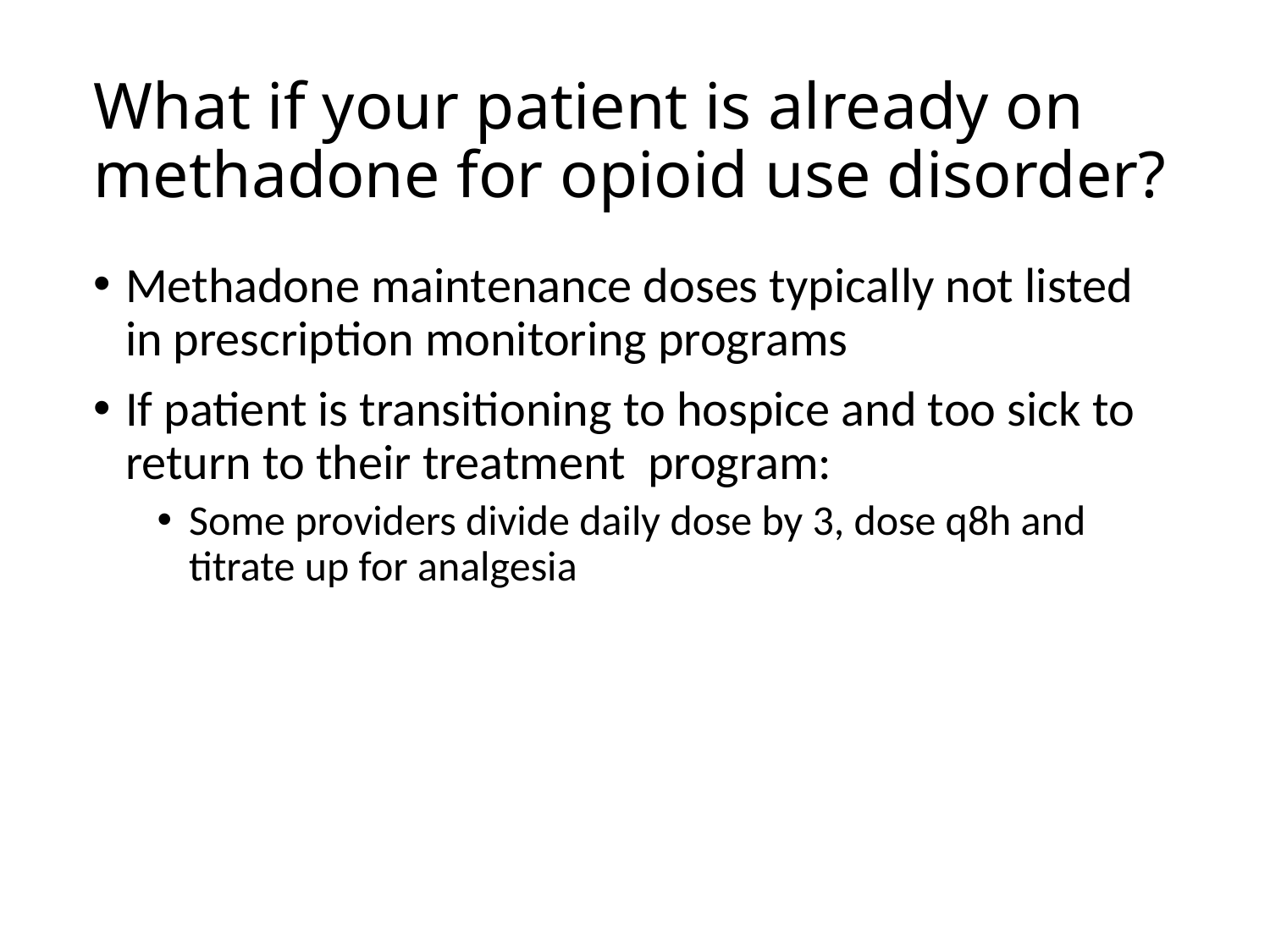

# What if your patient is already on methadone for opioid use disorder?
Methadone maintenance doses typically not listed in prescription monitoring programs
If patient is transitioning to hospice and too sick to return to their treatment  program:
Some providers divide daily dose by 3, dose q8h and titrate up for analgesia

## Slide 28
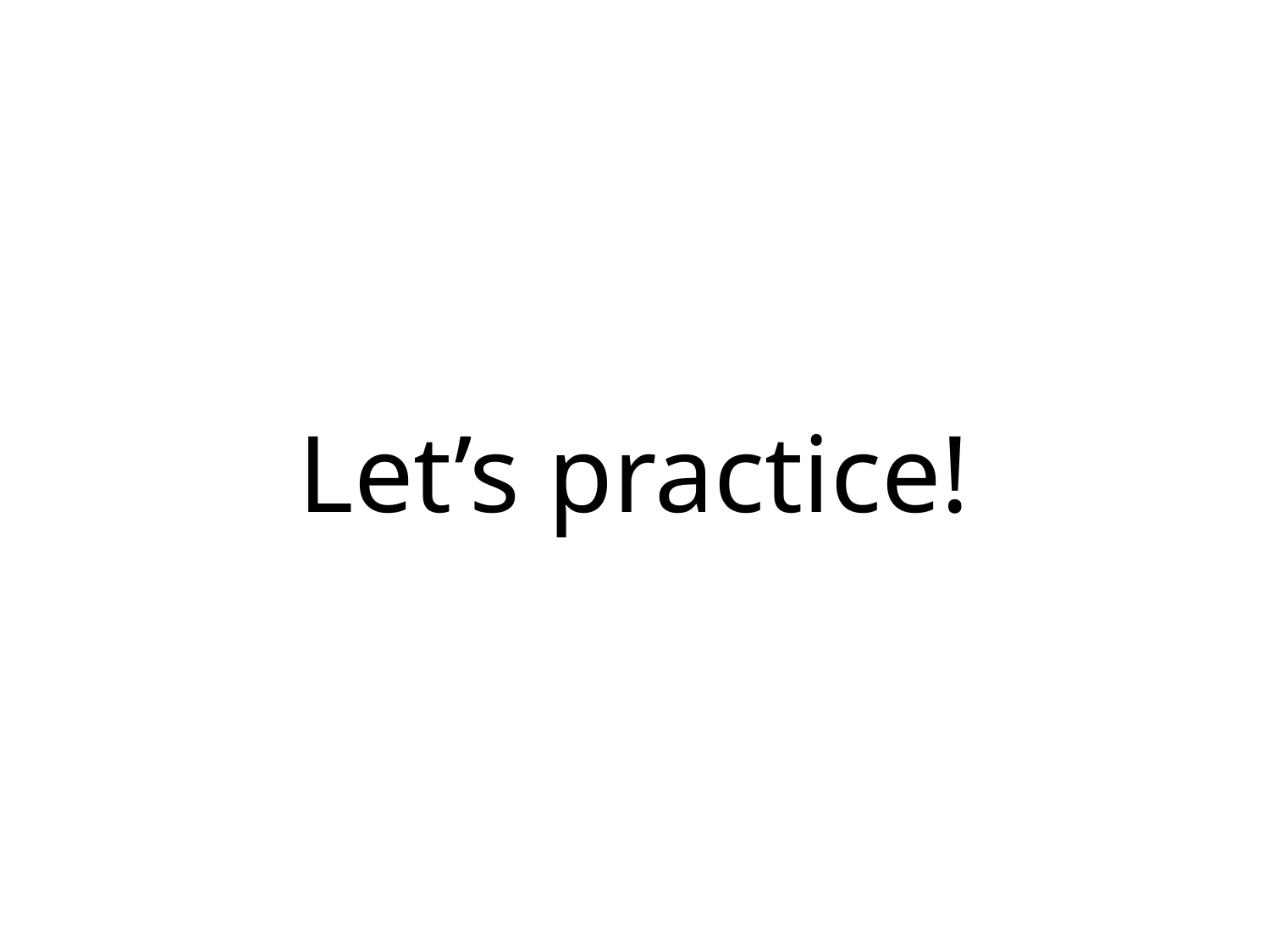

# Let’s practice!

## Slide 29
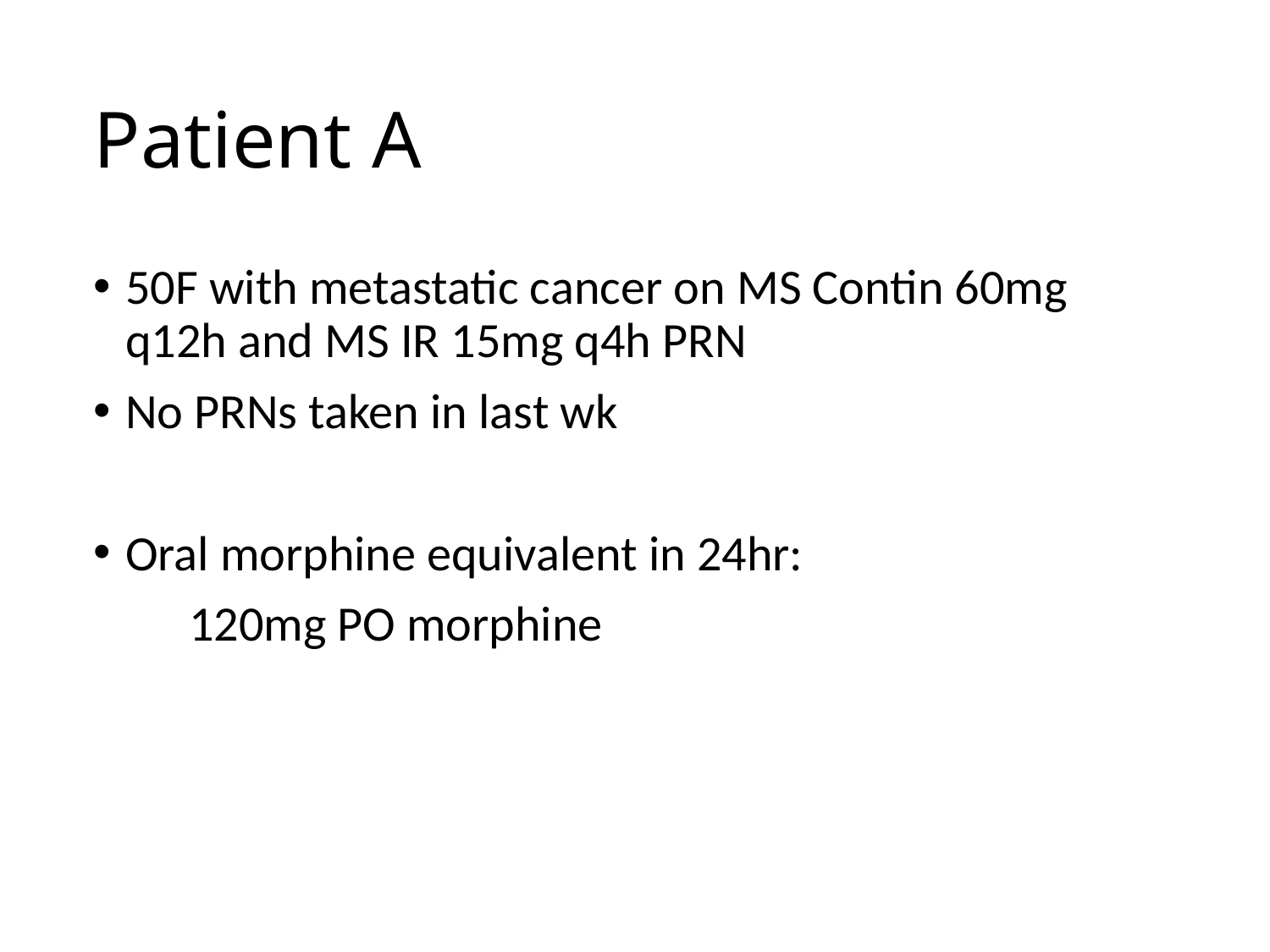

# Patient A
50F with metastatic cancer on MS Contin 60mg q12h and MS IR 15mg q4h PRN
No PRNs taken in last wk
Oral morphine equivalent in 24hr:
120mg PO morphine

## Slide 30
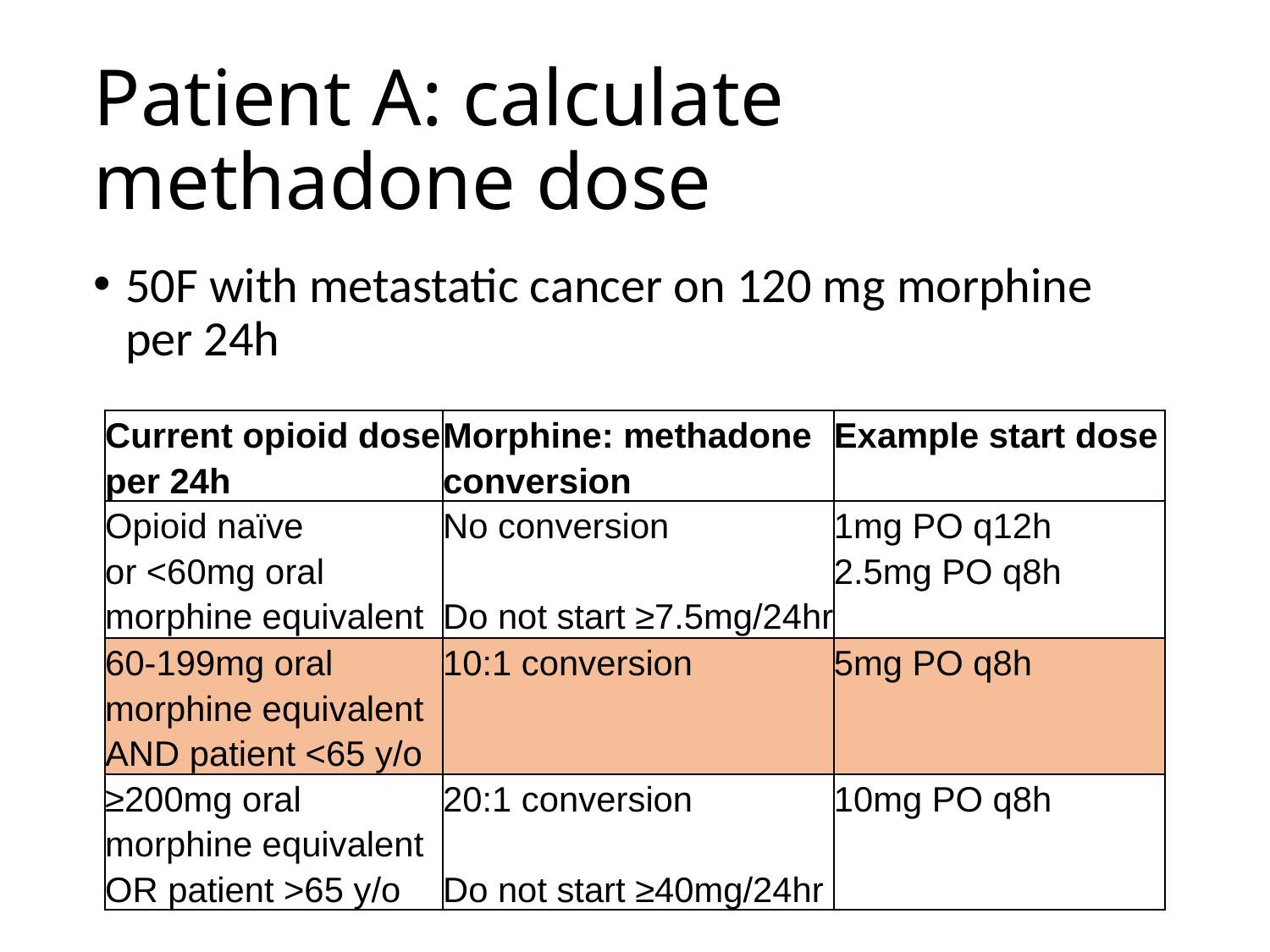

# Patient A: calculate methadone dose
50F with metastatic cancer on 120 mg morphine per 24h
| Current opioid dose per 24h | Morphine: methadone conversion | Example start dose |
| --- | --- | --- |
| Opioid naïve or <60mg oral morphine equivalent | No conversion   Do not start ≥7.5mg/24hr | 1mg PO q12h 2.5mg PO q8h |
| 60-199mg oral morphine equivalent AND patient <65 y/o | 10:1 conversion | 5mg PO q8h |
| ≥200mg oral morphine equivalent OR patient >65 y/o | 20:1 conversion   Do not start ≥40mg/24hr | 10mg PO q8h |

## Slide 31
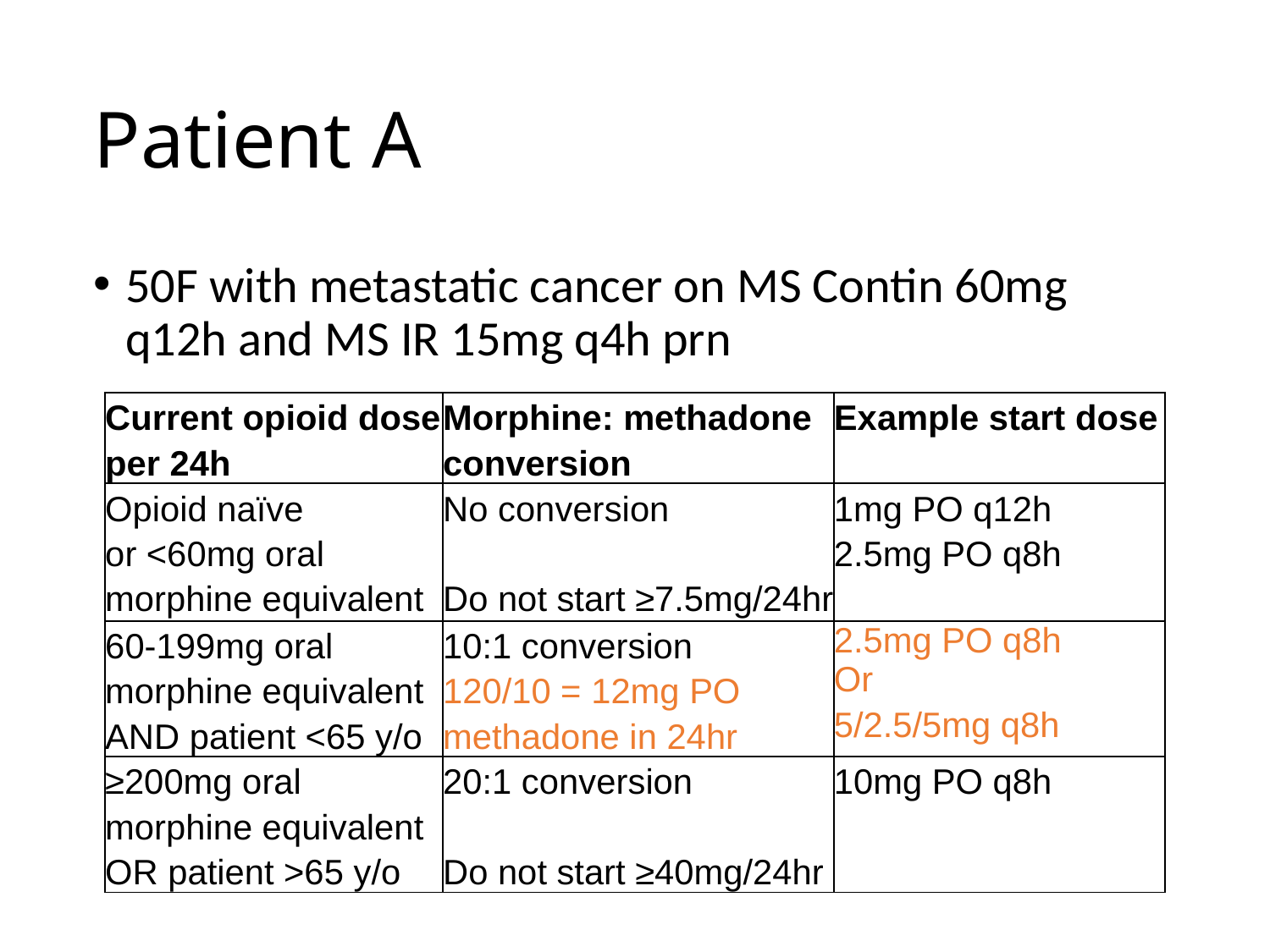

# Patient A
50F with metastatic cancer on MS Contin 60mg q12h and MS IR 15mg q4h prn
| Current opioid dose per 24h | Morphine: methadone conversion | Example start dose |
| --- | --- | --- |
| Opioid naïve or <60mg oral morphine equivalent | No conversion   Do not start ≥7.5mg/24hr | 1mg PO q12h 2.5mg PO q8h |
| 60-199mg oral morphine equivalent AND patient <65 y/o | 10:1 conversion 120/10 = 12mg PO methadone in 24hr | 2.5mg PO q8h Or 5/2.5/5mg q8h |
| ≥200mg oral morphine equivalent OR patient >65 y/o | 20:1 conversion   Do not start ≥40mg/24hr | 10mg PO q8h |

## Slide 32
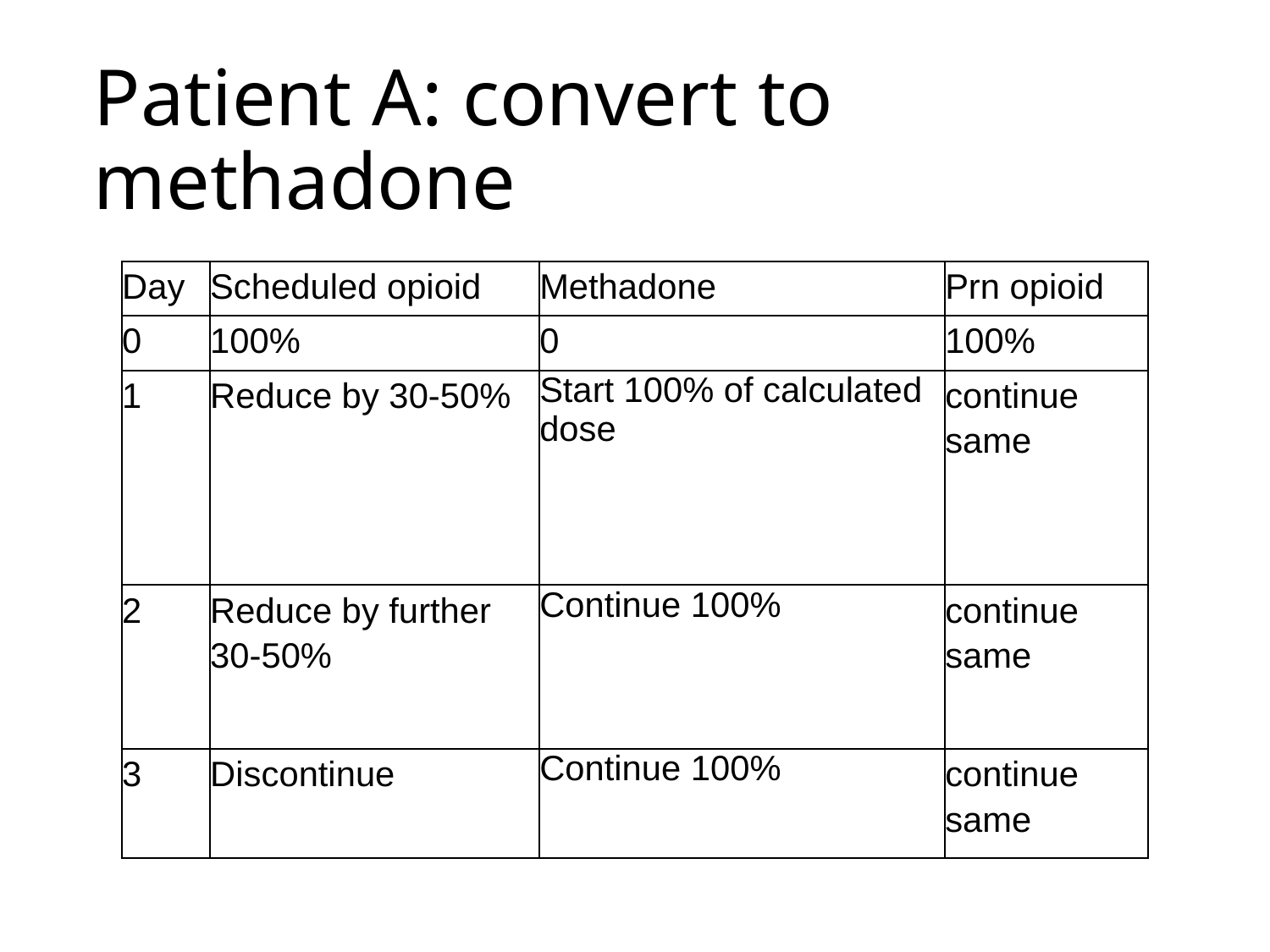

# Patient A: convert to methadone
| Day | Scheduled opioid | Methadone | Prn opioid |
| --- | --- | --- | --- |
| 0 | 100% | 0 | 100% |
| 1 | Reduce by 30-50% | Start 100% of calculated dose | continue same |
| 2 | Reduce by further 30-50% | Continue 100% | continue same |
| 3 | Discontinue | Continue 100% | continue same |

## Slide 33
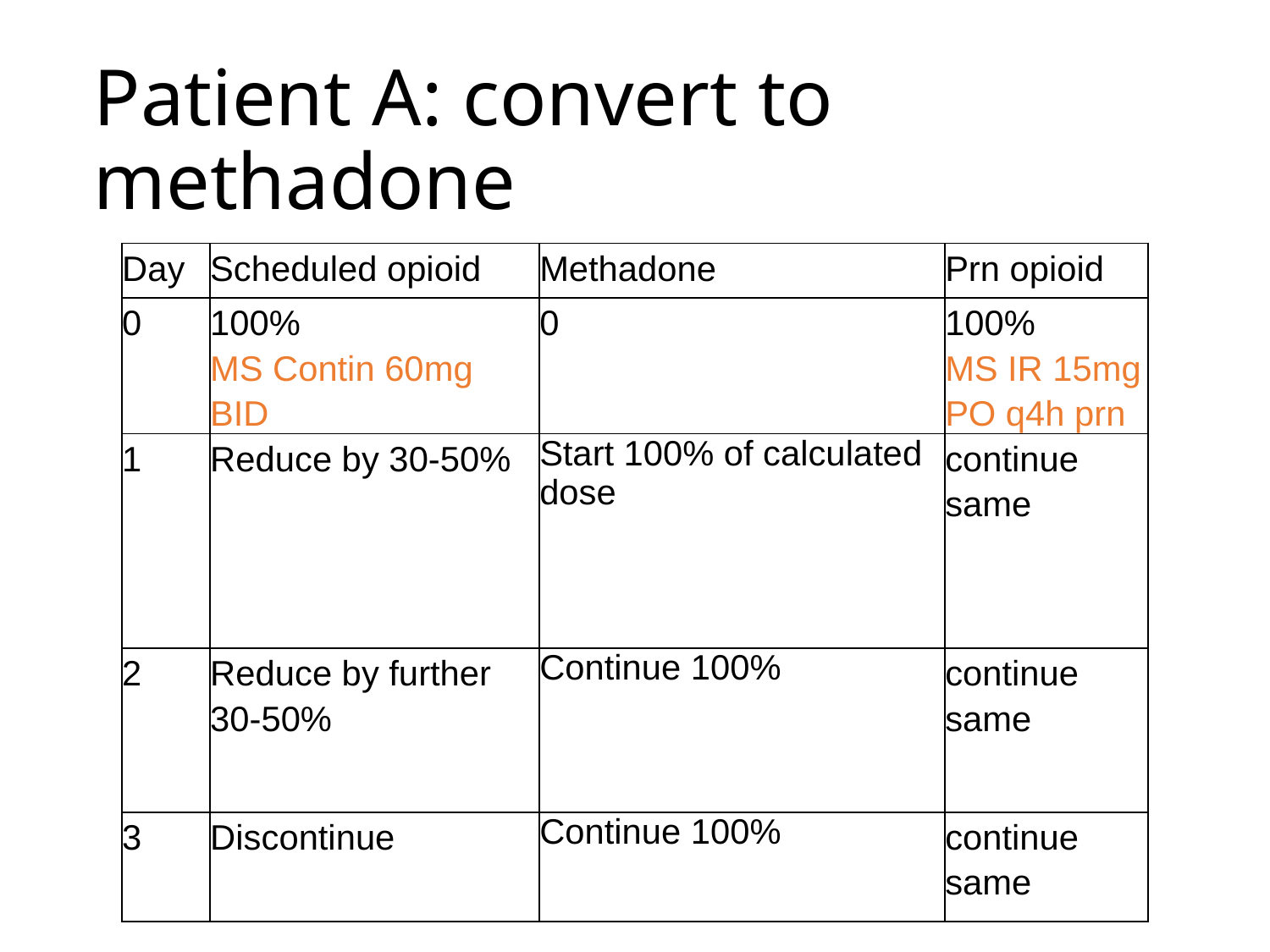

# Patient A: convert to methadone
| Day | Scheduled opioid | Methadone | Prn opioid |
| --- | --- | --- | --- |
| 0 | 100% MS Contin 60mg BID | 0 | 100% MS IR 15mg PO q4h prn |
| 1 | Reduce by 30-50% | Start 100% of calculated dose | continue same |
| 2 | Reduce by further 30-50% | Continue 100% | continue same |
| 3 | Discontinue | Continue 100% | continue same |

## Slide 34
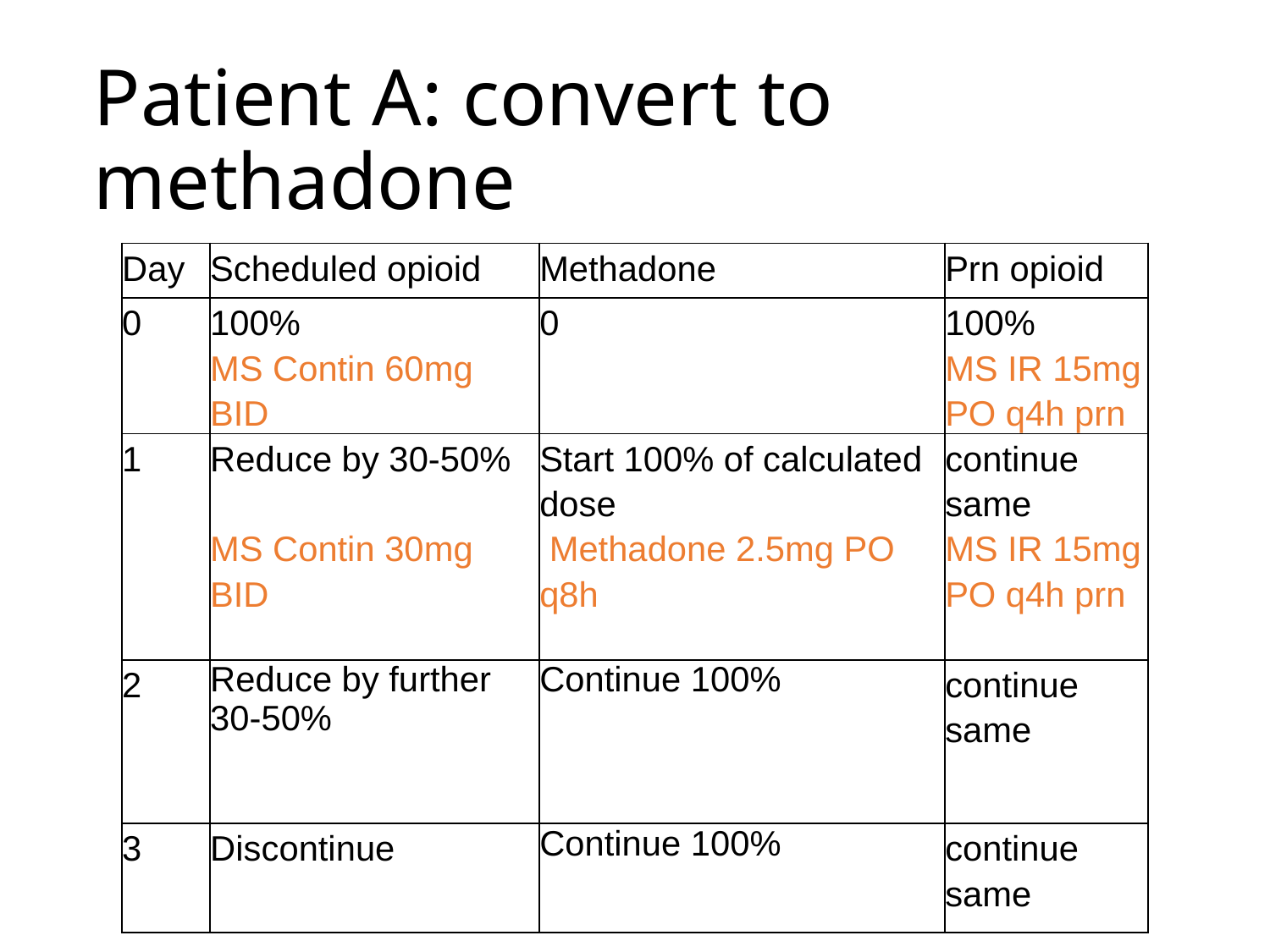

# Patient A: convert to methadone
| Day | Scheduled opioid | Methadone | Prn opioid |
| --- | --- | --- | --- |
| 0 | 100% MS Contin 60mg BID | 0 | 100% MS IR 15mg PO q4h prn |
| 1 | Reduce by 30-50% MS Contin 30mg BID | Start 100% of calculated dose Methadone 2.5mg PO q8h | continue same MS IR 15mg PO q4h prn |
| 2 | Reduce by further 30-50% | Continue 100% | continue same |
| 3 | Discontinue | Continue 100% | continue same |

## Slide 35
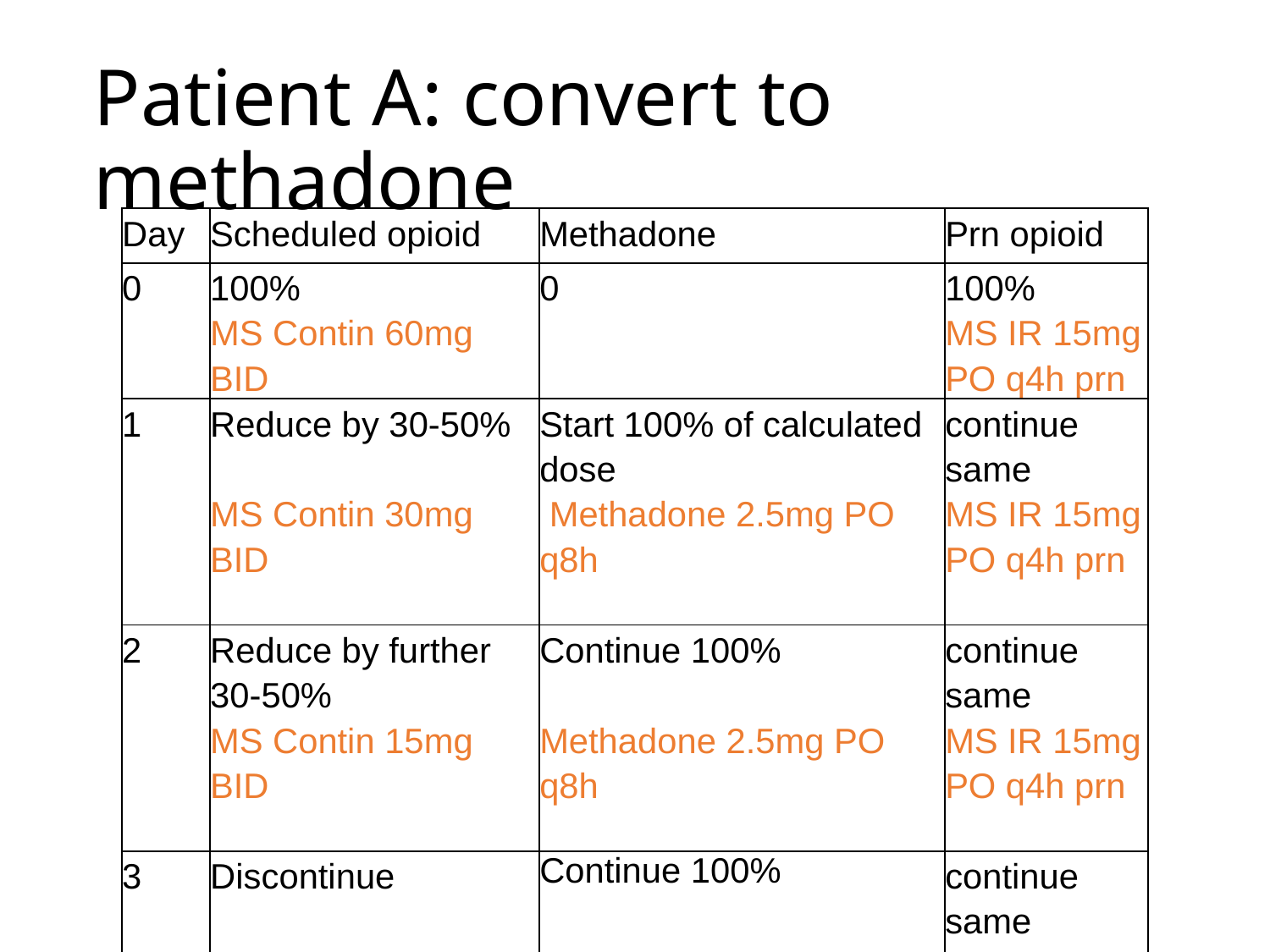

# Patient A: convert to methadone
| Day | Scheduled opioid | Methadone | Prn opioid |
| --- | --- | --- | --- |
| 0 | 100% MS Contin 60mg BID | 0 | 100% MS IR 15mg PO q4h prn |
| 1 | Reduce by 30-50% MS Contin 30mg BID | Start 100% of calculated dose Methadone 2.5mg PO q8h | continue same MS IR 15mg PO q4h prn |
| 2 | Reduce by further 30-50% MS Contin 15mg BID | Continue 100% Methadone 2.5mg PO q8h | continue same MS IR 15mg PO q4h prn |
| 3 | Discontinue | Continue 100% | continue same |

## Slide 36
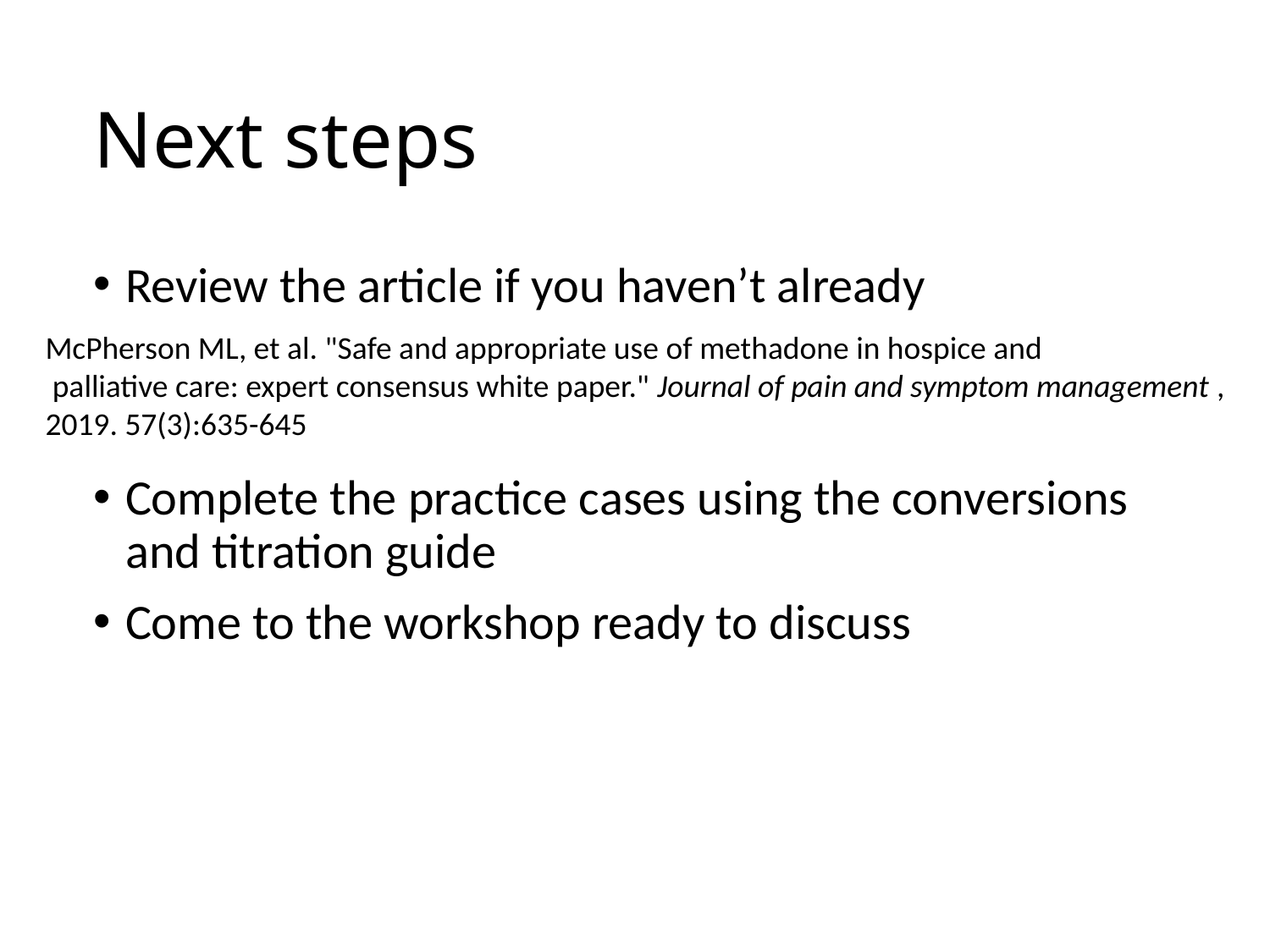

# Next steps
Review the article if you haven’t already
Complete the practice cases using the conversions and titration guide
Come to the workshop ready to discuss
McPherson ML, et al. "Safe and appropriate use of methadone in hospice and
 palliative care: expert consensus white paper." Journal of pain and symptom management ,
2019. 57(3):635-645
