# Supplementary material for: Methadone for Palliative Care Providers: A Case-Based Flipped Classroom Module for Faculty and Fellows
Source: MedEdPORTAL. 2021 Jul 26;17:11172. doi: 10.15766/mep_2374-8265.11172 (PMC8310899; doi:10.15766/mep_2374-8265.11172)
Supplement: Supplementary file 1 — Methadone Pretest.docxMethadone for Palliative Providers Slides.pptxMethadone Conversions and Titration Card.pdfMethadone Cases.docxMethadone Cases Teaching Guide.docxMethadone Posttest.docxMethadone Posttest Answer Key.docx [file mep_2374-8265.11172-s001.zip › C. Methadone Conversions and Titration Card.pdf]

Biewald 4.7.2021

How to start METHADONE for pain

CALCULATE METHADONE DOSE

Current opioid dose per 24h	Morphine: methadone conversion	Example start dose
Opioid naïve or <60mg oral morphine equivalent	No conversion<br><br>Do not start ≥7.5mg/24hr	1mg PO q12h<br>2.5mg PO q8h
60-199mg oral morphine equivalent AND patient <65 y/o	10:1 conversion	5mg PO q8h
≥200mg oral morphine equivalent OR patient >65 y/o	20:1 conversion<br><br>**Do not start ≥40mg/24hr**	10mg PO q8h

Note: table accounts for incomplete cross tolerance

Dose increases:
Generally do not increase methadone before day 5-7 (average steady state)
Increase by max 5mg/day for patients taking up to 40mg total/day
Increase by max 10mg/day for patients taking 40mg/day or more

Conversions:
from oral methadone to IV methadone: 2:1 ratio
Convert from IV methadone to oral methadone: 1:1.3 ratio
Convert from oral methadone to oral morphine approx. 1:3 ratio

EKGs:

- If goals of care are curative/life prolonging:

Check EKG prior to starting. If QTc 450-500 discuss risk/benefit with patient; if >500 consider other options. Repeat EKG within 2-4 weeks of starting, then again when total daily dose reaches 30mg and again when reaches 100mg.

- If goals are moderate

consider baseline EKG based on pt risk and preferences, and consider repeating as above

- If goals are fully comfort focused

no routine EKG unless there is a compelling reason to

Interactions:
Enzyme inhibitors slow methadone metabolism (amiodarone, cipro, sertraline)
Enzyme inducers increase methadone metabolism (HIV meds, carbamazepine)

CONVERT TO METHADONE

Day	Scheduled opioid	Methadone	Prn opioid	Note
0	100%	0	100%	Day 0 represents the opioid regimen **before** starting methadone
1	Reduce by 30-50%	Start 100% of calculated dose	continue same	On DAY 1, **begin methadone at full dose** divided into q8hr dosing. Decrease other scheduled opioid by 30-50%. Continue prn opioid
2	Reduce by additional 30-50%	Continue 100%	continue same	On DAY 2, continue current methadone dose and decrease other scheduled opioid by 30-50%
3	Discontinue	Continue 100%	continue same	On DAY 3, continue methadone and **discontinue scheduled opioid**

McPherson, Mary Lynn. Demystifying opioid conversion calculations: a guide for effective dosing (second edition). ASHP. 2018.
McPherson, ML, et al. Safe and Appropriate Use of Methadone in Hospice and Palliative Care: Expert Consensus White Paper. *JPSM.* 2019;57(3): 635-645.
