# Supplementary material for: Methadone for Palliative Care Providers: A Case-Based Flipped Classroom Module for Faculty and Fellows
Source: MedEdPORTAL. 2021 Jul 26;17:11172. doi: 10.15766/mep_2374-8265.11172 (PMC8310899; doi:10.15766/mep_2374-8265.11172)
Supplement: Supplementary file 1 — Methadone Pretest.docxMethadone for Palliative Providers Slides.pptxMethadone Conversions and Titration Card.pdfMethadone Cases.docxMethadone Cases Teaching Guide.docxMethadone Posttest.docxMethadone Posttest Answer Key.docx [file mep_2374-8265.11172-s001.zip › D. Methadone Cases.docx]

**MS. A**

Ms. A is a 75F with triple negative breast cancer metastatic to bone on chemotherapy who was hospitalized with pain. Her pain is constant, 9/10 in severity and has neuropathic and nociceptive qualities. Prior to admission she used morphine IR 30mg PO q4h prn for pain and frequently self-titrated her dose, using up to 10 prns/day. She also has severe anxiety for which she takes clonazepam 1mg BID. She also uses CPAP at night for apnea. She lives alone without assistance and her HCP is a son who lives out of state. Her son tells you that lately she seems forgetful.

- What characteristics make this patient an appropriate candidate for methadone therapy?

- What characteristics do not?

**MR. B**

Mr. B is a 40M with ESRD on HD and recently diagnosed head and neck CA who was hospitalized for trach/PEG placement and managed on a hydromorphone PCA for pain. The primary team wants you to suggest an opioid regimen for discharge. In the past 24h he used 600mg of IV hydromorphone. He tried a fentanyl patch once before and had a skin reaction. He has a history of IV heroin use. He lives in a small town and after completing cancer treatment at Mount Sinai wants to resume care with his PCP.

- What characteristics make this patient an appropriate candidate for methadone therapy?
- What characteristics do not?

**MR. C**

Mr. C is a 63M with multiple myeloma admitted with a new pathologic spine fracture and extensive osteolytic lesions. You have been working to manage his pain in the hospital. He is now comfortable on a regimen of morphine IR 30mg q4h scheduled and 15mg PO q2h prn. He used 4 prn doses in the past day.

- Calculate a methadone start dose for Mr. C. Try calculating the dose with and without including the prns.
- Create a conversion schedule for Mr. C.

| Day | Scheduled opioid | Methadone | Prn opioid |
| --- | --- | --- | --- |
| 0 |  |  |  |
| 1 |  |  |  |
| 2 |  |  |  |
| 3 |  |  |  |

- If his goals are life prolonging, at what points would you check an EKG?

- If he is going home with hospice and comfort focused goals and wants to minimize interactions with the health system, at what points would you check an EKG?

- His pain remains moderately controlled on your initial regimen. At what point would you recommend increasing the dose? By how many milligrams would you increase his dose?

**MS. D**

Ms. D is a 29F with a large retroperitoneal soft tissue sarcoma metastatic to the lung who was transferred from the oncology service to the palliative care unit for pain control. She is currently on a morphine PCA at 12mg per hour continuous/6mg IV q10min demand/24mg IV q1h clinician administered bolus. In the past 24hr she used 5 patient demand doses and no clinician boluses. Her goal is to get off the PCA and go home.

- Propose a methadone start dose and conversion schedule for Ms. D.

| Day | Scheduled opioid | Methadone | Prn opioid |
| --- | --- | --- | --- |
| 0 |  |  |  |
| 1 |  |  |  |
| 2 |  |  |  |
| 3 |  |  |  |

- Your conversion is successful and Ms. D is planning to return home with hospice when she develops esophageal candidiasis and can no longer swallow. Propose a conversion to IV methadone.
- A few weeks later, Ms. D can swallow again and wants to go home. Convert her back to PO methadone.

**MR. E**

Mr. E is a 90M with advanced COPD and lung cancer admitted to the hospital with pain due to a vertebral compression fracture. His pain is moderately controlled on morphine 7.5mg PO q4h prn and he has been using 3-4 doses per day.

- Propose a methadone start dose and conversion schedule for Mr. E.

| Day | Scheduled opioid | Methadone | Prn opioid |
| --- | --- | --- | --- |
| 0 |  |  |  |
| 1 |  |  |  |
| 2 |  |  |  |
| 3 |  |  |  |

- A week later Mr. E is doing well on his new methadone regimen. The intern pages you that Mr. E vomited after a dose of methadone and she is not sure what to. What would you advise her?
- Mr. E has a hospital course complicated by atrial fibrillation and a UTI. How might changes in his medical treatment affect his methadone level?
