# Supplementary material for: Methadone for Palliative Care Providers: A Case-Based Flipped Classroom Module for Faculty and Fellows
Source: MedEdPORTAL. 2021 Jul 26;17:11172. doi: 10.15766/mep_2374-8265.11172 (PMC8310899; doi:10.15766/mep_2374-8265.11172)
Supplement: Supplementary file 1 — Methadone Pretest.docxMethadone for Palliative Providers Slides.pptxMethadone Conversions and Titration Card.pdfMethadone Cases.docxMethadone Cases Teaching Guide.docxMethadone Posttest.docxMethadone Posttest Answer Key.docx [file mep_2374-8265.11172-s001.zip › E. Methadone Cases Teaching Guide.docx]

Objectives:

Select appropriate candidates for methadone therapy based on comorbidities and psychosocial issues

Calculate starting doses of methadone based on a patient’s prior opioid use

Choose degree of cardiac monitoring based on prognosis and goals of care

**MS. A**

Ms. A is a 75F with triple negative breast cancer metastatic to bone on chemotherapy who was hospitalized with pain. Her pain is constant, 9/10 in severity and has neuropathic and nociceptive qualities. Prior to admission she used morphine IR 30mg PO q4h prn for pain and frequently self-titrated her dose, using up to 10 prns/day. She also has severe anxiety for which she takes clonazepam 1mg BID. She also uses CPAP at night for apnea. She lives alone without assistance and her HCP is a son who lives out of state. Her son tells you that lately she seems forgetful.

- What characteristics make this patient a good candidate for methadone therapy?

*Severe pain, neuropathic quality, needs a long acting medication*

- What characteristics do not?

*Self titrating meds, no reliable caregiver, forgetful, sleep apnea with benzodiazepine use,*

**MR. B**

Mr. B is a 40M with ESRD on HD and recently diagnosed head and neck CA who was hospitalized for trach/PEG placement and managed on a hydromorphone PCA for pain. The primary team wants you to suggest an opioid regimen for discharge. In the past 24h he used 600mg of IV hydromorphone. He tried a fentanyl patch once before and had a skin reaction. He has a history of IV heroin use. He lives in a small town and after completing cancer treatment at Mount Sinai wants to resume care with his PCP.

- What characteristics make this patient a good candidate for methadone therapy?

*Intolerant of other opioids, needing high dose of opioid, PEG, no contraindication in renal failure, needs long acting med*

- What characteristics do not?

*History of opioid abuse, may not have a provider comfortable with prescribing methadone after discharge*

**MR. C**

Mr. C is a 63M with multiple myeloma admitted with a new pathologic spine fracture and extensive osteolytic lesions. You have been working to manage his pain in the hospital. He is now comfortable on a regimen of morphine IR 30mg q4h scheduled and 15mg PO q2h prn. He used 4 prn doses in the past day.

- Calculate a methadone start dose for Mr. C.

*If you don’t include the PRNs:*

*Morphine 30mg PO x 6 doses in 24 hr=180mg PO morphine*

*Select 10:1 conversion as pt’s current opioid dose is 60-199mg oral morphine equivalent and pt is <65 y/o*

*180mg PO morphine/10=18 mg PO methadone in 24 hr*

*Divide into q8h dosing: 18mg/3= 6mg. Closest dose that can easily be administered is 5mg tab*

*If you DO include the prns:*

*Morphine 30mg PO x 6 doses in 24 hr=180mg PO morphine*

*Morphine 15mg PO x 4 doses in 24 hr= 60mg PO morphine*

*Total: 180mg + 60mg = 240mg PO morphine*

*Select 20:1 conversion as pt’s current opioid dose is >200mg oral morphine equivalent and pt is <65 y/o*

*240mg PO morphine/20=12mg PO methadone in 24 hr*

*Divide into q8h dosing: 12mg/3= 4mg. Closest dose that can easily be administered is 5mg tab*

*This is an example of how prn use can push a conversion into a higher conversion ratio*

- Create a conversion schedule for Mr. C.

| Day | Scheduled opioid | Methadone | Prn opioid |
| --- | --- | --- | --- |
| 0 | *Morphine 30mg PO q4h scheduled* | *0* | *Morphine 15mg PO q2h prn* |
| 1 | *Morphine 15mg PO q4h scheduled (decrease by 1/2)* | *5mg PO q8h scheduled* | *“* |
| 2 | *Morphine 7.5mg PO q4h scheduled (decrease by further 1/2)* | *5mg PO q8h scheduled* | *“* |
| 3 | *0* | *5mg PO q8h scheduled* | *“* |

- If his goals are life prolonging, at what points would you check an EKG?

*Obtain baseline EKG prior to starting*

*Follow APS guidelines: repeat by 2-4 wks*

- If he is going home with hospice and comfort focused goals and wants to minimize interactions with the health system, at what points would you check an EKG?

*Follow low level of vigilance guidelines: no routine EKG*

- His pain remains moderately controlled on your initial regimen. At what point would you recommend increasing the dose? By how many milligrams would you increase his dose?

*Wait until methadone reaches steady state; average is 5-6 days*

*Increase by a maximum of 5mg/day up to total 40mg; increase by max 10mg/day beyond 40mg*

**MS. D**

Ms. D is a 29F with a large retroperitoneal soft tissue sarcoma metastatic to the lung who was transferred from the oncology service to the palliative care unit for pain control. She is currently on a morphine PCA at 12mg per hour continuous/6mg IV q10min demand/24mg IV q1h clinician administered bolus. In the past 24hr she used 5 patient demand doses and no clinician boluses. Her goal is to get off the PCA and go home.

- Propose a methadone start dose and conversion schedule for Ms. D.

*12mg IV morphine per hour x24h= 288mg IV morphine*

*Convert from IV to oral morphine: 288 x3=864mg PO morphine*

*Given that pt is using >200mg PO morphine per day, select 20:1 conversion ratio*

**note that because pt is using well over 300mg PO morphine daily, you may want to do a slower taper off of the PCA, and run your conversion by an experienced provider—this is a very conservative conversion.*

*864mg PO morphine/20= 43.2mg PO methadone in 24hr*

*Divide into q8h dosing: 43.2/3=14.4*

*Note: JPSM guidelines recommend never starting more than 30-40mg of total methadone per day. Based on this, reasonable options would be methadone 10mg TID, or 15/10/15*

| Day | Scheduled opioid | Methadone | Prn opioid |
| --- | --- | --- | --- |
| 0 | *Morphine 12mg IV per hour continuous* | *10mg q8h scheduled* | *Morphine 24mg IV q1h prn* |
| 1 | *Morphine 8mg IV per hour continuous (decrease by 1/3 of original dose)* | *“* | *“* |
| 2 | *4mg (decrease by further 50%)* | *“* | *“* |
| 3 | *Discontinue* | *“* | *“* |

- Your conversion is successful and Ms. D is planning to return home with hospice when she develops esophageal candidiasis and can no longer swallow. Propose a conversion to IV methadone.

*To convert from oral to IV methadone, use a 2:1 ratio*

*30mg PO methadone per 24hr /2= 15mg IV methadone per 24h*

*Divide into TID dosing: 15mg IV methadone/3= 5mg IV methadone q8h scheduled*

- A few weeks later, Ms. D can swallow again and wants to go home. Convert her back to PO methadone.

*To convert from IV to oral methadone, use a 1:1.3 ratio*

*15mg IV methadone per 24hr x 1.3 = 19.5mg PO methadone*

*Divide into TID dosing: 19.5mg PO methadone/3=6.5mg PO methadone q8h scheduled*

*Closest doses to 6.5mg are 5mg and 7.5mg*

*Note that the range of bioavailability is wide, hence the conversions between IV/PO and PO/IV are not identical*

**MR. E**

Mr. E is a 90M with advanced COPD and lung cancer admitted to the hospital with pain due to a vertebral compression fracture. His pain is moderately controlled on morphine 7.5mg PO q4h prn and he has been using 3-4 doses per day.

- Propose a methadone start dose and conversion schedule for Mr. E.

*Mr. E is using morphine 7.5mg PO x 4 doses per day= 30mg per day*

*Because he is using <60mg oral morphine per day, there is no conversion and he is considered essentially opioid naïve. Do not start >7.5mg PO methadone in 24hr. Example start doses include 1mg PO q12h if you are able to obtain liquid methadone, or 2.5mg PO q8 or q12h*

| Day | Scheduled opioid | Methadone | Prn opioid |
| --- | --- | --- | --- |
| 0 | *none* | *1mg PO q12h* | *7.5mg PO morphine q4h prn* |
| 1 | *“* | *“* | *“* |
| 2 | *“* | *“* | *“* |
| 3 | *“* | *“* | *“* |

- A week later Mr. E is doing well on his new methadone regimen. The intern pages you that Mr. E vomited after a dose of methadone and she is not sure what to. What would you advise her?

*If he vomited* *within the past 15min, replace full dose*

*Within 15-30min, replace half of dose*

*>30min do not replace dose*

- Mr. E has a hospital course complicated by atrial fibrillation and a UTI. How might changes in his medical treatment affect his methadone level?

*He may be taking amiodarone or an antibiotic such as ciprofloxacin. Both of these are enzyme inhibitors, meaning they will slow metabolism of methadone and result in an increased serum level. Remember to ask about the 3 As: amiodarone, anti-infectives, antidepressants.*
