# Supplementary material for: Methadone for Palliative Care Providers: A Case-Based Flipped Classroom Module for Faculty and Fellows
Source: MedEdPORTAL. 2021 Jul 26;17:11172. doi: 10.15766/mep_2374-8265.11172 (PMC8310899; doi:10.15766/mep_2374-8265.11172)
Supplement: Supplementary file 1 — Methadone Pretest.docxMethadone for Palliative Providers Slides.pptxMethadone Conversions and Titration Card.pdfMethadone Cases.docxMethadone Cases Teaching Guide.docxMethadone Posttest.docxMethadone Posttest Answer Key.docx [file mep_2374-8265.11172-s001.zip › G. Methadone Posttest Answer Key.docx]

Methadone posttest

Ms. D is a 50F with breast cancer metastatic to the spine and femur.  She takes MS contin 300mg BID and morphine immediate release 60mg q4h prn.  She has not taken any prns in a few days.  You want to convert Ms. D to methadone.  What is the highest starting dose you would prescribe?

1mg PO q12h

2.5mg PO q8h

5mg PO q8h

**10mg PO q8h**

*First calculate 24h total opioid usage. Ms. D used long acting morphine 300mg x2 and zero prn doses.*

*300mg PO morphine x2 = 600mg PO morphine in 24hr*

*refer to the “Calculate Methadone Dose” table in Appendix C*

*600mg PO morphine falls in the highest category, a 20:1 conversion to PO methadone*

*600mg/20=30mg PO methadone*

*30mg PO methadone/3 for q8hr dosing=10mg PO q8h*

Mr. R is an 89M with multiple myeloma who has diffuse pain due to widespread lytic lesions.  He takes long acting oxycodone 10mg BID and oxycodone 5mg immediate release q4h prn.  He took 2 prns in the past day.  Convert Mr. R to methadone.  What is the highest starting dose you would consider?

1mg PO q12h

**2.5mg PO q8h**

5mg PO q8h

10mg PO q8h

*First calculate 24 hr total opioid usage. Mr. R used long acting oxycodone 10mg x2 and oxycodone 5mg x2, for a total of 50mg oxycodone in 24 hr. Convert this to morphine using an equianalgesic table:*

*oxycodone 50mg PO (morphine 30mg PO/oxycodone 20mg PO)=75mg PO morphine*

*refer to the “Calculate Methadone Dose” table in Appendix C*

*75mg PO morphine falls in the middle category, a 10:1 conversion to methadone*

*75mg/10=7.5mg PO methadone in 24 hr*

*7.5/3 for q8h dosing = 2.5mg PO methadone q8h*

*Note that using this table, it is not necessary to reduce for incomplete cross tolerance*

Ms. G is a 30F with cervical cancer who is being treated on the palliative care unit with methadone 5mg PO q8h.  She can no longer tolerate PO.  What is the equivalent IV methadone regimen?

1mg IV q8h

**2.5mg IV q8h**

5mg IV q8h

10mg IV q8h

*Refer to the “Conversions” section in Appendix C*

*Oral methadone to IV methadone has an approximately 2:1 ratio*

*Methadone 5mg PO/2=2.5mg*

For a patient with life prolonging goals being treated with methadone for analgesia, when should you get an EKG to assess QTc?

Prior to starting

After 2-4 weeks

When the dose reaches 30mg total/24hr

When the dose reaches 100mg total/24hr

**All of the above**

*Refer to the “EKGs” section in Appendix C:*

*For a patient who has life prolonging goals, follow the American Pain Society guidelines for EKG monitoring, including assessing QTc prior to starting methadone, after 2-4wk on methadone, again if/when the total daily dose reaches 30mg, and again if/when the total daily dose reaches 100mg.*

Which comorbidity is **not** a contraindication to prescribing methadone for analgesia?

**ESRD on hemodialysis**

Congenital prolonged QTc

Recent IV heroin use

Sleep apnea and benzodiazepine use

*Patients on dialysis can be safely treated with methadone for pain.*

*Contraindications for methadone use include QTc prolongation, substance abuse, and concurrent sleep apnea with benzodiazepine use.*
